# Reassessment of HIV-1 Acute Phase Infectivity: Accounting for Heterogeneity and Study Design with Simulated Cohorts

**DOI:** 10.1371/journal.pmed.1001801

**Published:** 2015-03-17

**Authors:** Steve E. Bellan, Jonathan Dushoff, Alison P. Galvani, Lauren Ancel Meyers

**Affiliations:** 1 Center for Computational Biology and Bioinformatics, The University of Texas at Austin, Austin, Texas, United States of America; 2 Department of Biology, McMaster University, Hamilton, Ontario, Canada; 3 Yale School of Public Health, Yale University, New Haven, Connecticut, United States of America; 4 Department of Ecology and Evolution, Yale University, New Haven, Connecticut, United States of America; 5 Department of Integrative Biology, The University of Texas at Austin, Austin, Texas, United States of America; 6 The Santa Fe Institute, Santa Fe, New Mexico, United States of America; University of Southampton, UNITED KINGDOM

## Abstract

**Background:**

The infectivity of the HIV-1 acute phase has been directly measured only once, from a retrospectively identified cohort of serodiscordant heterosexual couples in Rakai, Uganda. Analyses of this cohort underlie the widespread view that the acute phase is highly infectious, even more so than would be predicted from its elevated viral load, and that transmission occurring shortly after infection may therefore compromise interventions that rely on diagnosis and treatment, such as antiretroviral treatment as prevention (TasP). Here, we re-estimate the duration and relative infectivity of the acute phase, while accounting for several possible sources of bias in published estimates, including the retrospective cohort exclusion criteria and unmeasured heterogeneity in risk.

**Methods and Findings:**

We estimated acute phase infectivity using two approaches. First, we combined viral load trajectories and viral load-infectivity relationships to estimate infectivity trajectories over the course of infection, under the assumption that elevated acute phase infectivity is caused by elevated viral load alone. Second, we estimated the relative hazard of transmission during the acute phase versus the chronic phase (RH_acute_) and the acute phase duration (*d*_acute_) by fitting a couples transmission model to the Rakai retrospective cohort using approximate Bayesian computation. Our model fit the data well and accounted for characteristics overlooked by previous analyses, including individual heterogeneity in infectiousness and susceptibility and the retrospective cohort's exclusion of couples that were recorded as serodiscordant only once before being censored by loss to follow-up, couple dissolution, or study termination. Finally, we replicated two highly cited analyses of the Rakai data on simulated data to identify biases underlying the discrepancies between previous estimates and our own.

From the Rakai data, we estimated RH_acute_ = 5.3 (95% credibility interval [95% CrI]: 0.79–57) and *d*_acute_ = 1.7 mo (95% CrI: 0.55–6.8). The wide credibility intervals reflect an inability to distinguish a long, mildly infectious acute phase from a short, highly infectious acute phase, given the 10-mo Rakai observation intervals. The total additional risk, measured as excess hazard-months attributable to the acute phase (EHM_acute_) can be estimated more precisely: EHM_acute_ = (RH_acute_ - 1) × *d*_acute_, and should be interpreted with respect to the 120 hazard-months generated by a constant untreated chronic phase infectivity over 10 y of infection. From the Rakai data, we estimated that EHM_acute_ = 8.4 (95% CrI: -0.27 to 64). This estimate is considerably lower than previously published estimates, and consistent with our independent estimate from viral load trajectories, 5.6 (95% confidence interval: 3.3–9.1). We found that previous overestimates likely stemmed from failure to account for risk heterogeneity and bias resulting from the retrospective cohort study design.

Our results reflect the interaction between the retrospective cohort exclusion criteria and high (47%) rates of censorship amongst incident serodiscordant couples in the Rakai study due to loss to follow-up, couple dissolution, or study termination. We estimated excess physiological infectivity during the acute phase from couples data, but not the proportion of transmission attributable to the acute phase, which would require data on the broader population's sexual network structure.

**Conclusions:**

Previous EHM_acute_ estimates relying on the Rakai retrospective cohort data range from 31 to 141. Our results indicate that these are substantial overestimates of HIV-1 acute phase infectivity, biased by unmodeled heterogeneity in transmission rates between couples and by inconsistent censoring. Elevated acute phase infectivity is therefore less likely to undermine TasP interventions than previously thought. Heterogeneity in infectiousness and susceptibility may still play an important role in intervention success and deserves attention in future analyses

## Abstract 2

Using simulated cohorts that account for previously unmeasured bias, Steve Bellan and colleagues provide new estimates of the duration and relative infectivity of the HIV-1 acute phase based on data from the retrospective cohort of serodiscordant couples in Rakai, Uganda.

## Editors' Summary

**Background.:**

About 35 million people are currently infected with HIV, the virus that causes AIDS, and more than 2 million people become newly infected with the virus every year, usually through having unprotected sex with an infected partner. Most people do not become ill immediately after infection, although some people develop a short flu-like illness. However, during this acute phase of infection, the amount of virus in the blood—the viral load—rises rapidly and peaks, before decreasing to a relatively stable lower level during the chronic phase of HIV infection. Chronic HIV infection, which may last for more than ten years, also has no major symptoms, but HIV slowly destroys immune system cells throughout this phase. Eventually, the immune system can no longer fight off infections by other disease-causing organisms, and HIV-positive people then develop one or more AIDS-defining conditions, including unusual infections and specific types of cancer; the HIV load also rises again during late phase infection.

**Why Was This Study Done?:**

Antiretroviral therapy (ART) can control, but not cure, HIV infection. By decreasing the viral load, ART not only improves the health of HIV-positive individuals, but also reduces their infectiousness. Consequently, experts believe that scaling up ART could substantially reduce the rate of new HIV infections. But the successful implementation of “treatment as prevention” faces several challenges. Notably, HIV testing and treatment programs need to be widely available, and people who are HIV-positive need to adhere to ART. Another major challenge that faces treatment as prevention is that HIV transmission that occurs during the acute phase of infection is likely to evade the intervention, and it is widely accepted that HIV-positive individuals are highly infectious during this phase of infection. However, acute phase infectivity has been directly measured only once: in a retrospectively identified group of serodiscordant heterosexual couples (couples in which only one partner was HIV-positive) in Rakai, Uganda. The authors of the current study found that existing estimates of acute phase infectivity failed to take account of important aspects of the Rakai study design or of heterogeneity (variability) in infectiousness or susceptibility among the study participants. Here, the researchers use mathematical modeling to compare simulated cohorts with the Rakai data to provide new estimates of the duration and relative infectivity of the acute phase that take into account study design and heterogeneity.

**What Did the Researchers Do and Find?:**

The researchers first used viral load trajectories and viral load–infectivity relationships to estimate infectivity trajectories over the course of infection. Using this approach, they estimated that the total additional risk attributable to the acute phase expressed as EHM_acute_ (excess hazard-months attributable to the acute phase of infection above the hazard generated by constant untreated chronic phase infectivity) was 5.6, which is considerably lower than previous estimates (which range from 31 to 141). Next, by fitting a mathematical model designed to simulate HIV infection and transmission within couples to the Rakai data, they estimated that the relative hazard of transmission during the acute phase versus the chronic phase (RH_acute_) was 5.3, that the acute phase duration (*d*_acute_) was 1.7 months, and that EHM_acute_ was 8.4. Finally, by replicating two highly cited analyses of the Rakai data on simulated data, the researchers show that the previous overestimates of acute phase infectivity likely stemmed from a failure to account for risk heterogeneity among study participants (some participants were more likely to transmit HIV or contract HIV than others because of underlying biological or behavioral differences in their infectiousness or susceptibility, respectively) and from bias arising from the retrospective cohort design of the Rakai study (serodiscordant couples who were lost to follow-up were excluded).

**What Do These Findings Mean?:**

In common with previous estimates of acute phase infectivity, the accuracy of these findings depends on the many assumptions made by the researchers in developing their mathematical models and on the quality of the data fed into these models. Nevertheless, these findings suggest that previous estimates of the infectivity of acute phase HIV infection are substantial overestimates. Thus, the researchers suggest, elevated infectiousness early in infection alone is unlikely to undermine treatment as prevention campaigns, and the population-level benefits of treatment as prevention may be larger than predicted from earlier estimates. These revised estimates—and the impact of heterogeneity of HIV infectiousness and susceptibility to infection on HIV transmission within populations revealed by this analysis—should now be considered when designing population-scale interventions and when communicating individual-level risk of HIV transmission and infection in clinical and community settings.

**Additional Information.:**

Please access these websites via the online version of this summary at http://dx.doi.org/10.1371/journal.pmed.1001801.

- This study is further discussed in a *PLOS Medicine* Perspective by Laith J. Abu-Raddad
- Information is available from the US National Institute of Allergy and Infectious Diseases on HIV infection and AIDS
- NAM/aidsmap provides basic information about HIV/AIDS, information about transmission and prevention, summaries of recent research findings on HIV care and treatment, and personal stories about living with AIDS/HIV
- Information is available from Avert, an international AIDS charity, on many aspects of HIV/AIDS, including detailed information on the stages of HIV infection and on treatment as prevention, and personal stories about living with HIV/AIDS
- The World Health Organization provides information on all aspects of HIV/AIDS (in several languages), including its guidelines on the use of ART for treating and preventing HIV infection
- The UNAIDS *World AIDS Day Report 2014* provides up-to-date information about the AIDS epidemic and efforts to halt it
- The *PLOS Medicine* Collection “Investigating the Impact of Treatment on New HIV Infections” provides more information about HIV treatment as prevention

## Introduction

Antiretroviral therapy (ART) reduces the infectiousness of HIV-infected individuals [1]. Both mathematical modeling and empirical research have suggested that scaling up antiretroviral treatment could substantially reduce the rate of new HIV infections [2,3]. However, there are numerous practical challenges for treatment as prevention (TasP) interventions, including broad implementation of HIV testing and treatment programs and ensuring adherence. In addition, HIV transmission immediately following infection may evade TasP if it occurs before infected persons are diagnosed, linked to care, and virally suppressed [4]. The success of TasP may therefore hinge on the fraction of HIV incidence attributable to transmission early after infection (AF_early_).

In general, HIV transmission depends on both sexual contact patterns and biological factors that influence the probability of infection per coital act, both of which can change throughout the course of infection. HIV viral load trajectories rise rapidly during the first few weeks following infection (acute phase) and then, after a cell-mediated host immune response, decrease to a relatively stable “viral set point” for many years (chronic phase), before rising again and leading to AIDS (late phase) [5]. These viral dynamics and the well-established relationship between viral load and infectivity [6,7] suggest that biological infectiousness is greatest during the acute phase, when viral load peaks. The enhanced acute phase infectivity is often characterized using two quantities: the relative hazard of transmission during the acute versus chronic phase (RH_acute_) and the acute phase duration (*d*_acute_). To clarify, “acute” refers to a period of elevated biological infectivity following infection, and “early” refers to a post-infection period (often longer than the acute phase) with a duration set by policy considerations (e.g., the lag between infection and first treatment) [4].

Acute phase infectivity may be even higher than expected based on viral load alone. Virion infectivity may decrease after the acute phase, for example because of viral evolution away from highly infectious strains that survive transmission bottlenecks or because of the accumulation of antibody coatings that reduce infectivity [8,9]. A macaque SIV experiment found that 7.5 to 750 times fewer virions were required to establish successful intravenous infection when the injected virus was derived from recently versus chronically infected macaques, suggesting that acutely infected animals may have higher per virion infectiousness [9]. However, we do not know whether HIV virion infectivity in humans is elevated during the acute phase and, if so, how quickly it declines to chronic phase levels.

The epidemiological implications of acute phase infectivity depend on the sociological context. In a serially monogamous population with long-lasting partnerships, elevated infectivity during the acute phase will contribute negligibly to transmission since acutely infected individuals will likely only re-expose the partner that infected them (unless they happen to change partners in that short period). In contrast, if partnerships are less stable, acutely infectious individuals may often expose new susceptible partners, and the acute phase may thereby contribute substantially to transmission. This will occur, for example, when there is a high prevalence of concurrent partnerships [10], when there is a generally fast partner switching rate, or if individuals exhibit episodic risk behavior (risk “volatility”) [11]. Nonlinear interactions between acute phase infectivity and patterns of sexual contact may increase AF_early_ far more than the sum of their separate effects [10,12]. Thus, it is necessary to understand acute phase infectivity in the context of sexual contact patterns to assess their joint contributions to transmission dynamics.

Although a high AF_early_ presents a challenge for TasP, it does not necessarily doom it to failure, because of an important trade-off between the timing and extent of transmission [13]. The observed exponential rise in HIV prevalence at the start of an epidemic can be explained by, at one extreme, infected individuals rapidly infecting a relatively small number of people (low *R*_0_ but high AF_early_) or, at the other extreme, infected individuals more slowly transmitting to many more people (high *R*_0_ but low AF_early_). In general, the amount of intervention required to contain an epidemic decreases with *R*_0_ [14]. Thus, early transmission (high AF_early_), which implies relatively low *R*_0_, makes early intervention more critical, but generally lowers the bar for success [4,13]. Consequently, some have proposed that the net effect of AF_early_ on the projected effectiveness of TasP interventions may be small [15], though this remains debated [16]. Even if our ability to control HIV transmission is not fundamentally limited by AF_early_, understanding the timing and magnitude of early transmission is critical for the design of cost-effectiveness interventions that maximally interrupt transmission.

However, estimates of AF_early_ range widely [4,8], depending on assumptions about RH_acute_, *d*_acute_, and sexual network characteristics. Here, we review the evidence for elevated acute phase infectivity, identify possible biases in widely accepted estimates of RH_acute_ and *d*_acute_, and reanalyze the available data to revise them accordingly.

Among 11 estimates of AF_early_ reviewed by Cohen et al. [8], all five studies focusing on sub-Saharan Africa used estimates of RH_acute_ and *d*_acute_ based on a retrospective cohort in Rakai, Uganda [17–22], which provides the only direct epidemiological measurement of acute phase infectivity and duration published to date. Of the remaining studies, none relied on a direct measure of acute phase infectivity. Instead, they considered the relationship between viral load trajectory and viral load infectivity [23], or relied on indirect estimates based on fitting a particular model to observed epidemic growth rates [24–29]. The lack of other data sources on newly infected HIV cases is not surprising. First, newly infected individuals are rarely tested within the short acute phase time window, and tests identifying recently infected individuals are not very reliable [8,30]. Second, newly infected individuals who are infected by their current sexual partner (who cannot be reinfected) provide no information on acute phase infectivity. Finally, ethics dictate that, when infected individuals with potentially susceptible partners are identified, interventions should be taken to prevent further transmission, so that identified individuals no longer transmit at rates reflective of the general population.

Given the influence of the Rakai study on the general understanding of the acute phase, we reanalyzed the results reported from this study to account for several previously overlooked sources of bias. Specifically, we fit a couples transmission model that accounted for the study design and for unmeasured transmission heterogeneity between couples. We then replicated previous analyses on simulated data to systematically explore the differences between our results, and those reported by the original study [17] and by the most widely cited reanalysis [18].

## Methods

S1 Text provides a complete model description and all scripts needed to reproduce our analyses. All analyses were performed in R [31].

### Excess Hazard-Months Attributable to Elevated Acute Infectivity

**Fig 1:**
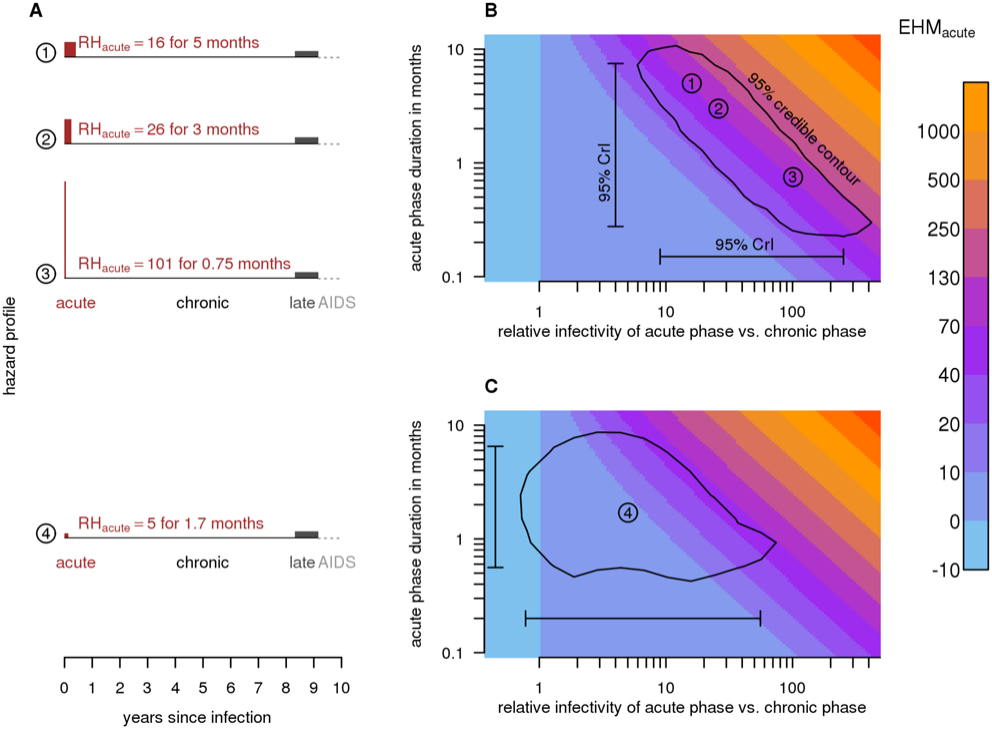
Excess hazard-months due to the acute phase. (A) Schematic diagram of relative infectiousness during HIV progression. In scenarios 1–3, the duration (*d*_acute_) and relative hazard of the acute phase (RH_acute_) differ; however, they all generate 75 excess hazard-months (EHM_acute_ = [RH_acute_ − 1] × *d*_acute_). The area of each acute phase rectangle (red; drawn to scale) represents the magnitude of EHM_acute_. Scenario 2 is the widely assumed acute phase infectivity that was estimated from the Rakai retrospective cohort using a variable hazard survival model [18]. Scenario 4 is our revised estimate obtained by fitting a couples transmission model to the same Rakai data (EHM_acute_ = 8.4). Unlike previous estimates, it accounts for unmodeled heterogeneity and the Rakai study’s exclusion criteria. (B) RH_acute_ versus *d*_acute_ for scenarios 1–3, along with 95% credibility intervals (95% CrIs) and a 95% credibility contour around estimates from the variable hazard survival model (scenario 2). Colors indicate EHM_acute_. Because couples in the Rakai cohort were observed at 10-mo intervals, the duration of the acute phase is not easily identified—shorter, highly infectious and longer, mildly infectious acute phases are both consistent with the data. EHM_acute_, however, can be estimated with greater relative precision. (C) Our best estimate of acute phase characteristics (scenario 4) and associated 95% CrI and credibility contour.

Previous studies focused on estimating RH_acute_, the relative hazard (i.e., infectivity) of the acute phase relative to the chronic phase. However, such estimates are not directly comparable across studies that assume (or estimate) different *d*_acute_ values (Fig. 1A and 1B). To overcome this limitation, we introduce a new measure: the excess hazard-months attributable to the acute phase (EHM_acute_), which equals (RH_acute_ − 1) × *d*_acute_. EHM_acute_ is defined, intuitively, in units of chronic phase hazard-months. If infectivity is constant throughout disease progression, then an infectious individual who dies 10 y after infection produces 120 hazard-months. If the acute phase is 3 mo long and 26 times as infectious as the chronic phase, then the acute phase contributes an additional EHM_acute_ = (26 − 1) ×3 mo = 75 hazard-months, for a total of 195 hazard-months. EHM_acute_ quantifies the total impact of *physiologically* elevated acute infectivity, and is comparable across studies. The overall contribution of each disease phase to population-level transmission is also influenced by sexual behavior (e.g., partner switching and concurrency). However, by focusing on data from stable couples, we separate the contribution of EHM_acute_ from that of partnership dynamics.

### Estimating EHM_acute_ from Viral Load

**Fig 2:**
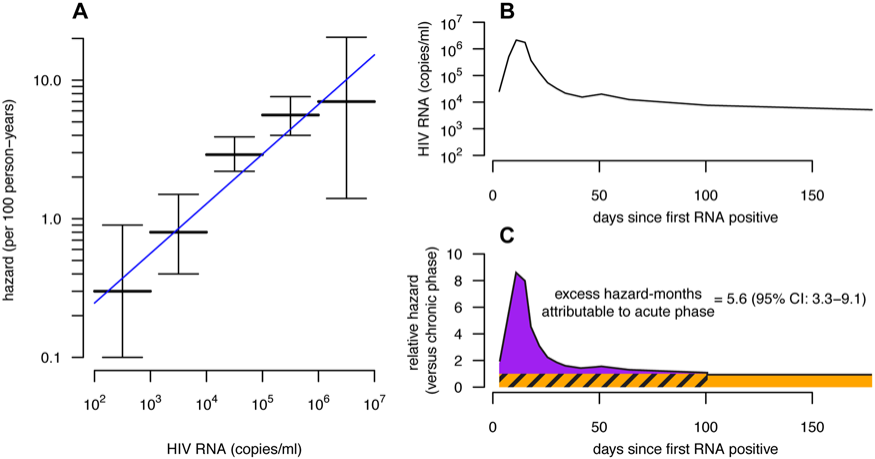
Viral-load-based estimates of excess hazard-months due to the acute phase. (A) The hazard of transmission by viral load category (horizontal bars with 95% confidence intervals [95% CIs]) from [7] with a fitted log-linear model (blue line). We compare these data to other studies in S7 Fig. (B) The average viral load trajectory of 19 recently infected individuals in East Africa from the ECHO cohort [32]. (C) Combining the fitted log-linear model (and 95% CIs on model coefficients) from (A) and the average viral load trajectory from (B), we estimated the relative hazard by disease phase (black line). The expected EHM_acute_ is the excess hazard-months occurring in the acute phase (area of the purple region), which can be compared with the baseline chronic hazard of equal duration (hatched orange area). While we drew the acute phase cutoff at 100 d based on the stabilization of the viral load near this time, it can be seen that EHM_acute_, because it is defined as excess hazard-months, is relatively insensitive to the cutoff time once the relative hazard approaches that of the chronic phase level (orange).

Published estimates of acute phase infectivity are believed to be higher than would be expected based on viral load alone [4]. However, viral load trajectories vary throughout the acute phase, increasing to a peak before declining to the chronic phase set point. If, as is commonly assumed, infectivity varies with viral load, then the instantaneous RH_acute_ also changes throughout the acute phase, and thus EHM_acute_ attributable to elevated acute phase viral load cannot be reliably inferred from snapshot estimates of RH_acute_ at the viral load peak [13]. Thus, we estimated the expected EHM_acute_ based on the viral load trajectory during the acute phase, rather than just the peak viral load. Combining empirical acute phase viral load trajectories [32] with a fitted log-linear model of infectivity as a function of viral load (with 95% CI [7]), we generated a relative hazard profile over an average disease progression, and summed the area under this profile to estimate EHM_acute_ caused solely by elevated acute phase viral load (Fig. 2).

### Couples Transmission Model

**Fig 3:**
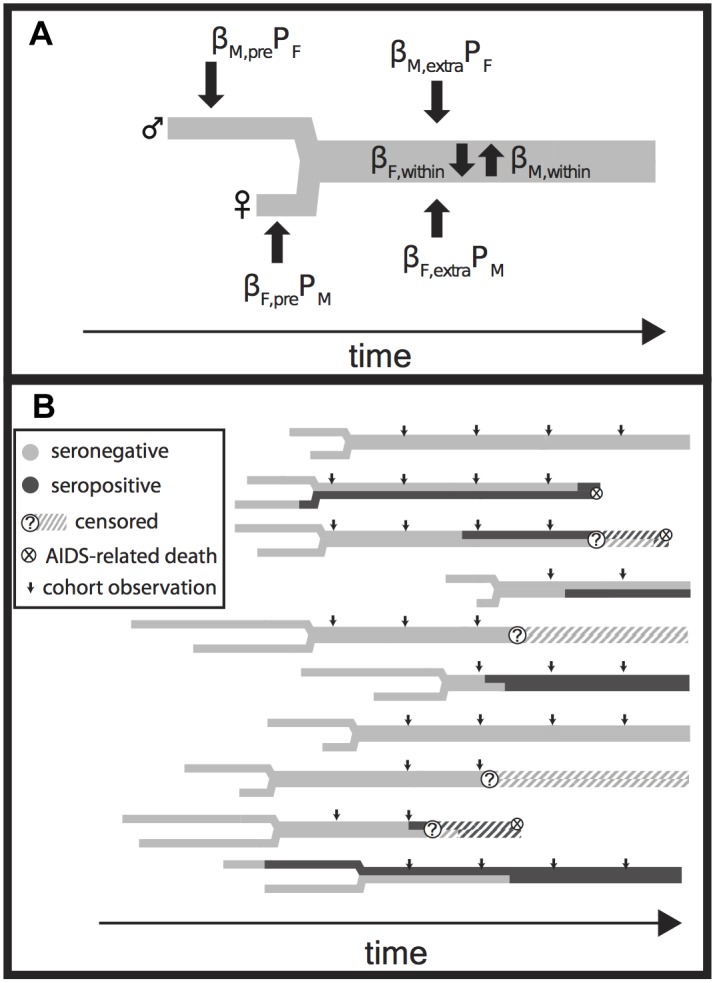
Model Diagram. (A) The relationship history of an example couple. Male (M, upper) and female (F, lower) branches begin at each partner’s sexual debut and then join together into a single thick gray line when they form a couple. Male and female partners are at risk of transmission prior to couple formation at a rate equal to the product of a transmission coefficient (β_M,pre_ and β_F,pre_) and the time-varying population prevalence in the opposite gender (P_F_ and P_M_). Transmission after the couple has formed from extra-couple partners is similarly dependent on the population prevalence. Infected individuals infect their stable partner at a rate equal to the product of a chronic phase transmission rate (β_M,within_ or β_F,within_) and the relative hazard of their current disease phase versus the chronic phase (not shown). Once infected, individuals are given Weibull distributed survival times [33,34] (not shown). (B) A simulated time series of infection, AIDS mortality, and censorship histories for ten couples. Small arrows indicate longitudinal observations of each couple, up to five times at 10-mo intervals if they have already formed at the start of observation, if they are not censored due to loss to follow-up or couple dissolution, and if both partners remain alive. These observations are then used to create a retrospective cohort (Fig. 4).

We adapted our previously published couples transmission model [33] for two purposes. First, we fit it to the Rakai retrospective cohort data to generate an independent estimate of EHM_acute_ (Fig. 3). Second, we used the model to simulate cohort data and thereby investigate discrepancies between prior estimates of EHM_acute_ and our own lower estimates.

**Fig 4:**
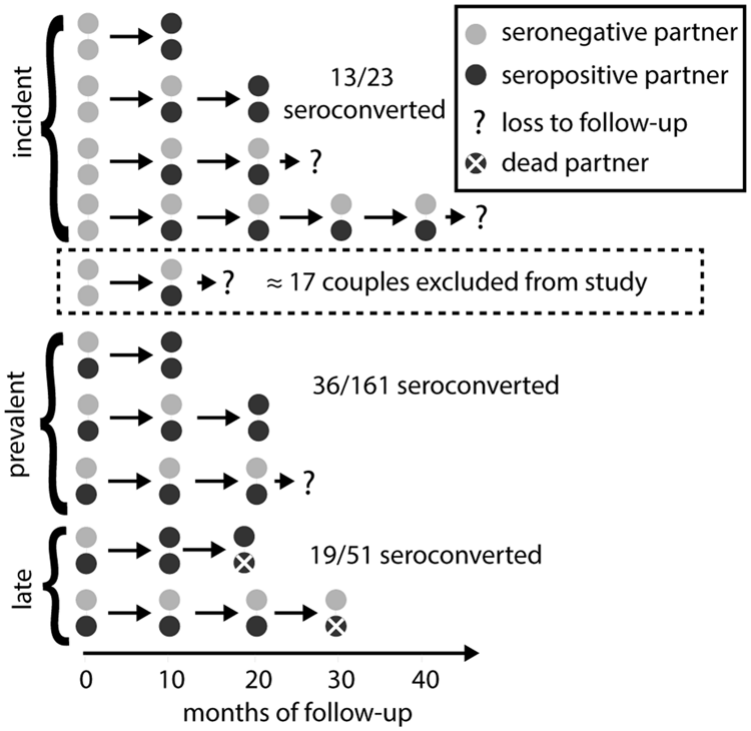
Rakai retrospective cohort study design. In both the original Rakai study and our simulated cohorts (Fig. 3), retrospectively identified serodiscordant couples (SDCs) were divided into those in which (1) the index partner’s infection occurred between study visits (incident SDCs), (2) the index partner’s infection occurred prior to study enrollment and neither partner died during follow-up (prevalent SDCs), and (3) the index partner’s infection occurred prior to study enrollment and the index partner died of AIDS during follow-up (late SDCs). Incident, prevalent, and late SDCs were assumed to reflect acute, chronic, and late phase infectivity exposure for the secondary partner (i.e., non-index partner). Couples recorded as serodiscordant only once and never seen again were excluded from the analysis under the assumption that these couples did not contribute any person-time at risk for transmission, while couples transitioning directly from concordant negative to concordant positive were included, whether or not they were subsequently observed. However, just as an immediate transition from concordant negative to concordant positive provides evidence for higher acute phase infectivity, a transition from concordant negative to serodiscordant provides evidence for lower acute phase infectivity. Thus, a sampling bias arises from this asymmetric exclusion of couples.

In the model, partners can be infected prior to couple formation, by a stable partner, or by an extra-couple partner while in a stable couple. We allowed the transmission rates between stable partners to vary according to the disease phase of the infected partner—acute, chronic, late, or AIDS. We also incorporated heterogeneity in risk by drawing individual hazards of infection from log-normal distributions with median ${\bar{\lambda }}_{\text{hazard}}$ and standard deviation σ_hazard_. We set uninformative uniform priors on acute phase parameters, median transmission rates, and σ_hazard_ (S1 Text). For each parameter set, we simulated a population of couples (see below), recording the timing of key events in disease progression (i.e., date of infection, death, and corresponding infection phases) and each individual’s hazard.

We constructed a “cohort” from the output of each simulation above according to the Rakai Community Cohort Study design [17]. Specifically, each couple’s serostatus was “observed” at 10-mo intervals from January 1994 through mid-1999. We then censored observations to simulate loss to follow-up and couple dissolution. Censorship was modeled as a serostatus-dependent process: couples that were concordant negative, serodiscordant, or incident serodiscordant (i.e., changed from concordant negative to serodiscordant between successive cohort observations) at a given cohort observation had a 25%, 35%, and 47% probability, respectively, of being censored before the subsequent cohort observation, reflecting empirically observed rates [35,36].

Using the criteria of Wawer et al. [17], we selected a “retrospective cohort” from each of these simulated “cohorts” that included all couples that were observed serodiscordant and then observed in at least one more subsequent visit, along with all couples that were observed concordant negative and then concordant positive at the subsequent visit. Importantly, these criteria exclude couples that were observed concordant negative and then serodiscordant only once before being censored by loss to follow-up, couple dissolution, or the end of the study (Fig. 4), under the assumption that these couples did not contribute any person-time at risk. However, each couple that transitions from concordant negative to serodiscordant provides evidence for lower acute phase infectivity, while each couple that transitions from concordant negative to concordant positive provides evidence for higher acute phase infectivity. The exclusion of the former but not the latter couples creates sampling bias. By including this sampling process in our model, we explicitly accounted for this bias.

Retrospective cohort couples were then classified as “incident,” “prevalent,” or “late,” depending on information about the stage of the index partner (i.e., first partner infected). Specifically, “incident” couples were those in which the index partner became infected while the couple was under cohort observation; the secondary partners (i.e., non-index partner) in such couples were therefore exposed to an acutely infected index partner during the observation period. Couples that were not incident were classified as “prevalent” unless the index partner was recorded as having died during the study, in which case they were classified as “late.” Late couples thus constitute couples in which the secondary partner was exposed to an index partner in the AIDS phase (too sick to infect), and possibly in the late phase (increased infectivity) preceding it.

The Rakai study used molecular viral linkage assays to identify seroconverting secondary partners who had been infected by an extra-couple partner, and to exclude them from the cohort. To replicate this in our fitted model, we similarly excluded all couples where the second partner was infected via extra-couple transmission. We conducted a sensitivity analysis to this exclusion in the simulation analysis below because such couples do contribute person-time at risk up until the secondary partner’s infection.

### Estimating EHM_acute_ from the Rakai Retrospective Cohort

We fit the couples transmission model to the Rakai retrospective cohort data using approximate Bayesian computation with sequential Monte Carlo (ABC-SMC) [37] to estimate transmission rates, RH_acute_, *d*_acute_, and σ_hazard_. We describe our approach in detail in S1 Text. Briefly, we used the model to simulate 4,875 couples (i.e., the Rakai couples cohort size [36]) for each of hundreds of thousands of parameter sets drawn from uninformative prior distributions (S1 Table). Parameter sets that generated retrospective cohorts sufficiently similar to the Rakai cohort, as measured by several summary statistics, were accepted, while others were rejected. Summary statistics included proportions of secondary partners seroconverting in incident and prevalent couples and the extent of individual heterogeneity as indicated by discrepancies between unadjusted and adjusted regression analyses (S2 Table). New parameter sets, sampled randomly around those accepted in the previous step, were simulated and then again filtered based on similarity to the Rakai data. This filtering procedure was repeated with increasingly strict criteria for similarity, until the distribution of parameters converged and the simulation summary statistics sufficiently matched the real data.

### Simulating Previous Estimates of EHM_acute_

To identify biases underlying discrepancies between our estimates and prior estimates from the Rakai cohort data, we replicated the two most highly cited analyses of the Rakai retrospective cohort (Poisson regression [17] and variable hazard survival model [18]) on simulated data across a wide range of parameter sets (S3 Table). We examined differences between estimated parameters and the “true” values used in the simulation. To reduce the effect of sampling error, we simulated cohorts of 100,000 couples in this analysis.

### Poisson Regression Model

We replicated the approach of Wawer et al. [17] and estimated RH_acute_ from each simulated retrospective cohort using a Poisson regression of secondary partner seroconversion against index partner disease phase (acute, chronic, late), controlling for secondary partner person-time at risk (as an offset term). Person-time at risk was calculated by assuming that infections or deaths occurring in a 10-mo interval occurred at the 5-mo midpoint of the interval. Similarly, when both partners were infected in the same interval, this analysis assumed that secondary partner infection occurred at 7.5 mo. For incident couples, only the interval in which the index partner seroconverted was considered representative of acute phase exposure for the secondary partner. Consequently, later observation intervals were excluded from the regression. Given the person-time assumptions above, this interval represents the secondary partner’s exposure to the first 0–5 mo of the index partner’s infection. Thus, this approach implicitly assumes that the acute phase lasts 5 mo.

In [17], observed covariates that potentially affect transmission (coital rate, genital ulcer disease [GUD], viral load, and age) were included in the regression. Adjusting for observed covariates controls for some, but not all, of the heterogeneity between couples because other sources of variation remain unobserved. We similarly simulated “observed covariates” that were partially correlated with each individual’s actual hazard and adjusted for these covariates. This allowed us to assess estimation accuracy under the assumption that the study included observed covariates that account for some but not all of the variance in risk between individuals.

### Variable Hazard Survival Model

Hollingsworth et al. [18] reanalyzed the Rakai retrospective cohort by fitting a variable hazard survival model to the data. Because their estimates of acute and late phase infectivity and duration are frequently considered the best available estimates [4,8,10,19,38,39], we also replicated their analysis on our simulated retrospective cohorts. This model explicitly specifies different hazards and durations for the acute, chronic, and late phases, and also specifies the existence of an AIDS phase prior to death during which no transmission occurs (Fig. 1A here and Fig. 1 in [18]). Rather than assuming a 5-mo acute phase as in [17], this approach explicitly estimates both phase infectivity and duration.

This model makes the following assumptions. Secondary partners in prevalent couples are exposed to a chronically infected index partner. Secondary partners in incident SDCs are exposed to an acutely infected index partner for a duration *d*_acute_ beginning when their partner was infected, and are exposed to a chronically infected index partner thereafter. Similarly, secondary partners in late SDCs cannot be infected during the AIDS phase preceding their partner’s death, are exposed to the late phase preceding that, and are exposed to the chronic phase preceding that. Rather than assuming that infections occurred at the midpoint of an interval, this model more realistically considered the timing of the index partner’s infection as an unknown, hidden event that occurred with equal prior probability at each time during the interval of occurrence.

Hollingsworth et al. estimated phase durations and hazards and 95% CIs using a maximum likelihood approach, but did not estimate confidence intervals for derived parameters such as RH_acute_ or EHM_acute_. We fitted the same model using a Bayesian Markov chain Monte Carlo algorithm, which better facilitated estimation of 95% CrIs (the Bayesian analogue of confidence intervals) for all parameters of interest. We validated our fitting procedure by fitting to data simulated by this same variable hazard survival model with known parameters, and assessing our ability to accurately recover these parameters.

## Results

We found that analyses of cohort data with 10-mo survey intervals cannot distinguish between shorter, highly infectious and longer, less infectious acute phases because of the relatively long intervals between couple observations. While collinearity between *d*_acute_ and RH_acute_ prevents these parameters from being identifiable, EHM_acute_ can be estimated with relatively greater precision (Fig. 1A and 1B). EHM_acute_ values can be compared to the 120 hazard-months an individual would produce over 10 y of infection if infectivity was constant. Thus, an EHM_acute_ of 12 would indicate that the acute phase contributes an additional 10% of the hazard that an individual would produce during 10 y of untreated chronic infection.

Based on viral load–infectivity relationships alone, we estimate that the hazard of transmission at peak viral load is approximately nine times greater than at the chronic phase set point. However, this peak is transient, and the viral load trajectory across the entire acute phase suggests an EHM_acute_ of only 5.6 (95% CI: 3.3–9.1) (Fig. 2).

**Fig 5:**
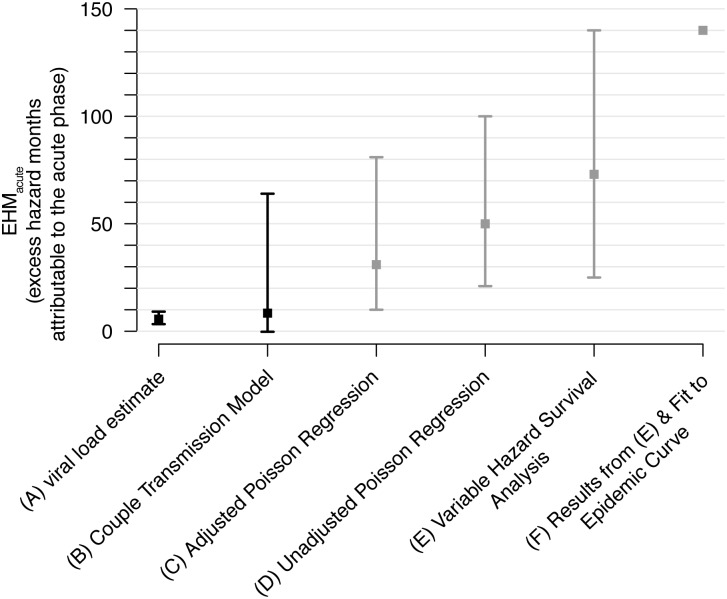
Revised acute phase estimates. Our estimates (black) of the excess hazard-months attributable to the acute phase (EHM_acute_) based on (A) viral load trajectories (Fig. 2) and (B) our fit of a couples transmission model to the Rakai retrospective cohort. We compare these estimates with previous Rakai-based estimates that did not adjust for these biases (gray). These include (C) Wawer et al.’s adjusted and (D) unadjusted Poisson regressions [17], (E) Hollingsworth et al.’s variable hazard survival analysis [18], and (F) Powers et al.’s estimates that used a Bayesian framework to combine estimates from (E) and a mathematical modeling fit to an epidemic curve [19].

We fit a couples transmission model that explicitly accounts for couple dissolution, loss to follow-up and the cohort exclusion criteria of the Rakai retrospective cohort data (Figs. 3 and 4) to estimate acute phase infectivity and duration, mean transmission rates into and between couples, and inter-individual heterogeneity in these transmission rates. Our model fit the data well (S1 Fig.). From this analysis, we estimated EHM_acute_ to be 8.4 (95% CrI: −0.27 to 63), RH_acute_ = 5.3 (95% CrI: 0.79–57), and *d*_acute_ = 1.7 mo (95% CrI: 0.55–6.8) (Figs. 1C and 5; Table 1). We estimated the median transmission rate between partners to be ${\bar{\lambda }}_{\text{hazard}}$ = 12 (95% CrI: 4.6–30) per 100 person-years and the heterogeneity in transmission to be σ_hazard_ = 2.0 (95% CrI: 1.2–2.8). A σ_hazard_ of 2.0 corresponds to individuals at the 97.5% highest risk quantile, experiencing transmission rates 50-fold greater than the median.

**Table 1:** Acute phase infectivity estimates from Rakai cohort data.

Model	Acute Hazard	Chronic Hazard	*d*_acute_ ^a^	RH_acute_	EHM_acute_	Notes
Poisson regression (unadjusted) [17]	0.0082 (per coital act)	0.0007 (per coital act)	5	11 (5.2–21)^b^	50^c^ (21–100)^d^	Unadjusted hazards (incident couple in first interval of observation versus all prevalent couple observations).
Poisson regression (adjusted for coital rates) [17]	—	—	5	8.3 (3.4–20)	36^c^ (12–96)^d^	Reduction in EHM_acute_ from the raw estimates indicates that greater hazards in incident couples were partly explained by higher coital rates in incident couples that seroconverted couples.
Poisson regression (adjusted for coital rates, age, and self-reported GUD) [17]	—	—	5	7.3 (3.1–17)	31^c^ (10–81)^d^	Further reduction in EHM_acute_ suggests that incident couples were younger and had greater co-infection rates than prevalent couples; these systematic differences contributed to their increased hazard relative to prevalent couples.
Variable hazard survival model [18]	2.8 (0.91–27)^e^ (per year)	0.11 (0.075–0.14) ^e^ (per year)	2.9 (0.29–7.4) ^e^	26 (8.1–270) ^e^	73 (24–150) ^e^	More realistic accounting for person-time than regression models above, but without controlling for any covariates indicative of heterogeneity.
Couples transmission model	**0.62^f^ (0.083–8.4)** (per year)	**0.12^f^ (0.046–0.30)** (per year)	**1.7 (0.55–6.8)**	**5.3 (0.79–57)**	**8.4^g^** (−0.27 to 64)	Estimates reduced when fully accounting for the study design and both observed and unobserved heterogeneity (S5 Table).

95% CIs (first three rows) or 95% CrIs (last two rows) in parentheses. Estimates of acute and chronic phase hazards, acute phase duration (*d*_acute_), the acute to chronic phase relative hazard (RH_acute_), and the excess hazard-months due to the acute phase (EHM_acute_) from the Rakai retrospective cohort study. Best estimates from our analysis are shown in bold.

^a^In months.

^b^Confidence interval calculated based on a Poisson regression without controlling for any covariates or coital rates.

^c^These EHM_acute_ values were calculated from relative hazards per coital act by assuming that these were approximately equal to relative hazards per unit time since coital rates in incident (10.2/mo) and prevalent (10.0/mo) couples were comparable.

^d^These confidence intervals assume a known acute phase duration of 5 mo.

^e^95% CrIs from our refit of the variable hazard survival model with Bayesian Markov chain Monte Carlo, which provided a more complete and accurate characterization of the uncertainty around these point estimates than the maximum likelihood estimation approach of the original analysis.

**Fig 6:**
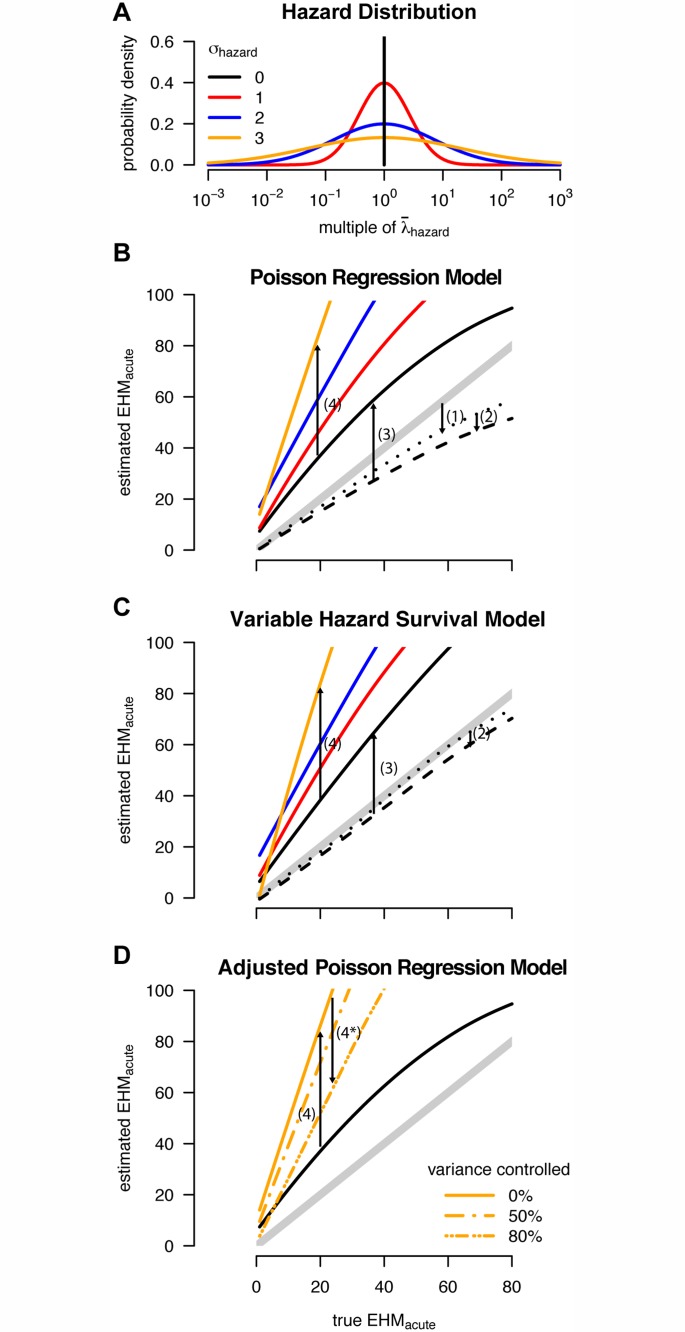
Multiple sources of bias for acute phase estimates. (A) The log-normal distributions used to model variability in individual hazard of infection (color-coding of σ_hazard_ used throughout the figure). (B and C) Estimated excess hazard-months attributable to the acute phase (EHM_acute_) versus the true (simulated) EHM_acute_ when analyzing simulated cohort data with the (B) Wawer et al. [17] Poisson regression and (C) Hollingsworth et al. [18] variable hazard survival model. Thick gray diagonal lines represent unbiased estimates. Arrows 1–4 indicate how each bias affects estimates of EHM_acute_. Arrow locations along the *x*-axis are chosen for ease of display only; for any true EHM_acute_, each bias is quantified by the vertical separations between lines. Dotted lines show the “best case” scenario for these models: if the underlying population is truly homogenous, the analysis includes all seroincident couples, and late and chronic phase infectivity are equal. The small downward bias (1) in the Poisson regression arises from assumptions regarding person-time at risk. The dashed lines reveal additional downward bias (2) in both models stemming from misclassification of late couples as prevalent couples (assuming the excess hazard-months attributable to the late phase was 40). Solid lines show estimates from simulated cohorts when seroincident couples lost to follow-up are excluded (Fig. 4), causing bias (3). Finally, both analyses are further biased upward (4) when used to analyze heterogeneous populations. (D) The same trends for σ_hazard_ = 0 and 3 from (B), but also showing how bias (4) can be partly mitigated (4*) when variance between individuals is controlled for by adjusting for measured covariates corresponding to some (but not all) of the heterogeneity. (B–D) were created by fitting smoothers through individual simulations (S3 Fig.).

^f^Median transmission hazards for a population of individuals experiencing log-normally distributed transmission hazards (Fig. 6A). These estimates should be interpreted within the context of this heterogeneity.

^g^The slight discrepancy between our median EHM_acute_ = 8.4 and (RH_acute_ − 1) × *d*_acute_ = (5.3 − 1) × 1.7 = 7.3 is because the median of a function is not necessarily equal to the function of its inputs’ medians.

We replicated previously published methods for estimating acute phase infectivity in simulated populations of 100,000 couples, yielding an average retrospective cohort size of 3,000 couples (i.e., after excluding couples with no infections or as dictated by the study’s exclusion criteria), which was sufficient to distinguish inherent biases from random fluctuations (S2 Fig.). We identified four sources of bias that influenced estimates of EHM_acute_ produced by either the Poisson regression [17] or variable hazard survival model [18] (Fig. 6B–6D). The first bias stems from assumptions about the timing of seroconversion between cohort observations. The Poisson regression approach assumed that the first infection in a given 10-mo observation interval occurred at the 5-mo midpoint. When an incident infection and secondary infection occurred in the same interval, this approach assumed that the latter occurred at 7.5 mo (i.e., the midpoint between the first infection and the end of the interval). In theory, the interacting effects of these three assumptions can be complex (S3 Fig.), but our simulations show that, on the whole, these assumptions led to a small downward bias in estimates of EHM_acute_. By assuming instead that infection events occurred at unknown times with equal prior probability distributed throughout an interval, the variable hazard survival model removed this bias.

The second bias stems from misclassification of some late phase couples as chronic phase couples when they are lost to follow-up just prior to a partner’s death. The accidental inclusion of some late phase couples leads to overestimates of chronic phase infectivity, which, in turn, biases estimates of RH_acute_ and EHM_acute_ downward (Figs. 6B, 6C, and S4). Both the regression and survival models were affected similarly by this small downward bias.

The last two biases caused overestimates of EHM_acute_ that far outweighed the first two downward biases (Fig. 6). Specifically, the exclusion of incident SDCs lost to follow-up (Fig. 4) nearly doubles estimates of EHM_acute_ relative to the true value. This bias is best illustrated with unadjusted hazard calculations. Assuming that index and secondary infections occurred at the 5-mo and 7.5-mo points within each study interval, we can calculate the acute phase hazard from the original data in which ten of 23 incident couples had both partners seroconvert in the same interval (S4 Table):

$$\beta_{\text{acute}}=\frac{10\text{ infections}}{10\;\times \;2.5\;+\;13\;\times \;5\text{ person-months}}=0.11\;\frac{\text{infections}}{\text{person-months}}$$ (1)

However, based on empirical rates of loss to follow-up in Rakai, it is likely that approximately 17 incident SDCs were excluded because they were not seen again after their incident serodiscordant visit, either because they dissolved, were subsequently lost to follow-up or because their incident serodiscordant visit coincided with the end of the study period [35,36]. If we correctly include these couples in our calculation, then the estimated acute phase hazard is much lower:

$$\beta_{\text{acute}}=\frac{10\text{ infections}}{10\;\times \;2.5\;+\;\left(13+17\right)\;\times \;5\text{ person-months}}=0.057\;\frac{\text{infections}}{\text{person-months}}$$ (2)

Our analysis finds that this effect approximately doubled previous estimates of acute phase transmission that relied on this retrospective cohort. We assumed 17 (43%) of incident SDCs were excluded from the original data for this illustrative example. However, in fitting our couples transmission model, we explicitly modeled this sampling procedure, and the proportion of incident SDCs excluded was variable between simulations fitted to the data and was driven by empirical estimates of the loss-to-follow-up and couple dissolution rates in Rakai. Our ABC-SMC fitted posterior median number of incident SDCs excluded was 17 (95% CrI: 8–35), corresponding to 43% (95% CrI: 27%–60%) of incident SDCs being excluded (S1 Text; S5 Fig.). Importantly, this uncertainty in the exact number excluded by the Rakai study is reflected by our new estimates of EHM_acute_.

The fourth bias emerges from unmodeled heterogeneity in the risk of transmission within SDCs. Couples with higher risk are, by definition, more likely to transmit infection. Thus, couples that have remained persistently serodiscordant (prevalent and late couples) represent couples with a lower transmission risk, on average, than newly formed “naïve” SDCs (incident couples). Thus, a portion of the estimated EHM_acute_ may actually reflect sampling-based differences between couples that enter the study serodiscordant versus seroconcordant negative, rather than biological differences between acute and chronic phase infectivity. The adjusted Poisson regression analysis partly corrects for this bias, by adjusting for covariates, which accounts for some of this heterogeneity (Fig. 6D), whereas the variable hazard survival analysis does not correct for any covariates. In the original regression analysis, adjustment for covariates reduced the estimated EHM_acute_ from 50 to 31. Inclusion of additional risk covariates, had they been measured, would have reduced this estimate further. By fitting our heterogeneous couples transmission model above, we explicitly accounted for this bias while estimating the extent of heterogeneity.

These four biases account for virtually all of the difference between the Poisson regression and variable hazard survival estimates and the true simulated values being estimated (S5 Table); thus, other remaining sources of bias are necessarily minor. In particular, the exclusion of couples in which the secondary partner was infected in an extra-couple partnership was not a substantial source of bias (S6 Fig.), in part because such couples were excluded from both incident and prevalent categories, and these effects approximately balanced each other.

## Discussion

In addition to reducing morbidity, ART also reduces the risk that HIV-infected individuals infect their sexual partners [1]. TasP has consequently become a primary focus of HIV control strategies [40]. Still, many have highlighted that TasP prevents transmission only from individuals who have been diagnosed and treated [19]. This has spawned an energetic debate concerning the proportion of transmission likely to occur too early after infection to be preventable by realistic TasP interventions [4]. In particular, the assumption that individuals are extremely infectious during the several months immediately following infection has led to arguments that a large proportion of all transmission occurs too early to be averted by TasP [4,19,39].

We have found that the evidence for elevated acute phase infectivity, a key component of early transmission, is not nearly as strong as commonly thought. Acute phase infectivity has been directly measured only once, from a retrospective couples cohort in Rakai [17]. We reanalyzed the reported results from this study, accounting for the sampling procedure and individual-level heterogeneity in the risk of HIV transmission, which were overlooked in all previous analyses. Our new estimate for the acute phase hazard is nine times less than the currently most frequently used estimate [8,18]. Thus, physiologically elevated infectiousness early in infection alone is unlikely to undermine TasP campaigns. Furthermore, intervention efforts targeted at identifying acutely infected individuals [41] may be less cost-effective at preventing forward transmission than previously thought.

In general, long intervals between observations may preclude precise estimation of shorter duration events. The 10-mo Rakai observation interval contributes substantial uncertainty to estimates of the duration (*d*_acute_) and relative hazard (RH_acute_) of the acute phase. A short, intense acute phase and a long, milder acute phase will exhibit similar transmission patterns at a 10-mo level of resolution. To circumvent this uncertainty, we introduced EHM_acute_, the excess chronic-phase-equivalent hazard-months attributable to elevated acute infectivity (EHM_acute_ = [RH_acute_ − 1] × *d*_acute_). Unlike RH_acute_, EHM_acute_ can be estimated from cohort studies, even when the duration of the acute phase is unknown. The magnitude of EHM_acute_ can be compared to the baseline of 120 chronic phase hazard-months untreated infected individuals would generate over their approximately 10 y of constant infectiousness if the acute and late phases had infectivity equal to that of the chronic phase. We estimated an EHM_acute_ of 8.4 (95% CrI: −0.27 to 64), lower than the original estimate of 31 and far lower than the currently most frequently used estimate of 73 [17,18]. We find that the upward revision from the first regression-based estimate of EHM_acute_ = 31 to the survival-analysis-based estimate of 73 was almost entirely attributable to the latter’s exclusion of covariates that the former used to captured some, but not all, of the heterogeneity in transmission risk. Furthermore, we showed that both previous estimates were biased upward by the study design itself and by additional unobserved heterogeneity in couple transmission rates.

The effect of controlling for heterogeneity can be seen in the original regression analysis [17]. Adjusting for coital rates reduced the estimated EHM_acute_ from 50 to 36 (Table 1), and adjusting for age and GUD further reduced the estimate to 31. Correcting for other sources of inter-couple heterogeneity (e.g., host and viral genotypes affecting susceptibility and infectiousness, tendency to use condoms, other co-infections) would have likely reduced the estimate even further.

Our findings demonstrate the utility of simulation approaches for validating epidemiological study design and analysis [42,43]. Bias may arise in unexpected ways from interactions between epidemiological, observation, and sampling processes. All of these processes can be included in simulation models that can be fit directly to empirical data with modern statistical approaches. Such models can also be used to simulate data for analysis to compare the performance of alternative methods. Comparisons between estimates from simulated data, where the underlying true parameters are known, provides a powerful tool with which to discover biases and evaluate the robustness of estimators when not all assumptions are met, or in the presence of sampling bias. For example, our initial aim was to examine the effect of heterogeneity on estimates of acute phase infectivity. Unexpectedly, replication of previous approaches on our homogenous simulations also yielded biased estimates, leading us to discover three other sources of bias.

Our two independent estimates of EHM_acute_—one based on elevated acute phase viral load and the other based on the Rakai data—were similar, with each inside the confidence bounds of the other. Thus, contrary to the prevailing consensus [8,18,19], we cannot reject the null hypothesis that elevated acute phase infectivity in humans is caused solely by the transient elevation in viral load (and not elevated per virion infectivity). However, we emphasize that the variance in all estimates based on this small cohort reflects considerable uncertainty, which should be propagated in all analyses of acute phase infectivity, particularly those calculating AF_early_. To that end, in addition to providing our estimates of EHM_acute_, RH_acute_, and *d*_acute_ and their credibility intervals above, we have provided our fitted posterior distribution of acute phase infectivity and duration and individual heterogeneity in transmission to facilitate future modeling work (S1 Data). We emphasize that models relying on these estimates of acute phase infectivity and duration should also adequately account for their collinearity (i.e., the upper confidence bounds of both RH_acute_ and *d*_acute_ are not, as a pair, within their joint credibility contour; Fig. 1) and also consider individual heterogeneity.

### The Acute Phase Debate

There is considerable disagreement regarding the impact of early transmission on the effectiveness of TasP [4,13,15,16]. Powers et al. estimated that the fraction of HIV incidence attributable to transmission from acutely infected individuals (AF_acute_) was 40% [19], while Williams et al. argued that AF_acute_ was more likely to be 2%–4% [44]. The discrepancy arises from the former’s confidence and the latter’s skepticism in the variable hazard survival analysis’s estimates of acute phase infectivity and duration from Rakai (Fig. 1B; [18]), which we show are upward-biased by unmodeled heterogeneity and study design (Figs. 1C and 6).

Powers et al. fit an HIV transmission model to antenatal clinic prevalence trends in Lilongwe, Malawi, using a Bayesian procedure to update prior estimates of acute phase infectivity from Rakai [19]. Their analysis provided posterior estimates of EHM_acute_ = 141, nearly double the estimate that formed the basis for their prior, and 16 times our best estimate. Their further inflation of EHM_acute_ stems from their fit to the Lilongwe epidemic, which (like many HIV epidemic trajectories) exhibits a steep initial rise in prevalence followed by deceleration to a lower epidemic peak than would be expected based on the initial rise. However, the observed steep epidemic growth in Lilongwe is largely driven by one antenatal clinic observation in 1987 with substantial uncertainty. More importantly, this characteristic epidemic trajectory can be explained by mechanisms other than high acute phase infectivity. For example, heterogeneity and assortativity in risk behavior can drive rapid early growth as HIV spreads through high-risk subpopulations [15,45]. Declining risk behavior over the course of the epidemic can also explain relatively rapid early growth [15,46]. Therefore, high estimates of EHM_acute_ derived by fitting to epidemic trajectories are unreliable.

Phylogenetic clustering of incident infections has also been used to infer the proportion of transmission attributable to early infection [47–49]. However, these studies make varying assumptions regarding the time window after infection considered “early,” which precludes direct comparison of the AF_early_ estimates. Furthermore, recent work has uncovered several questionable assumptions in phylogenetic and phylodynamic inference of transmission events [50–52] and has suggested that conclusions reached from these approaches should be interpreted cautiously. For example, phylogenetic tree topologies may not correspond to transmission networks, sampled individuals are not unbiased random samples of infected individuals, several viral genotypes may be transmitted during infection, and certain genotypes may be preferentially transmitted.

In addition, AF_early_ estimates may be strongly influenced by the intervention history in the focal population. Large AF_early_ values are often interpreted as an obstacle to future TasP success, but they could instead indicate ongoing TasP success. Successful TasP will decrease transmission following the initiation of treatment, thereby increasing the *relative* transmission rate of the pretreatment period (i.e., AF_early_). As increasingly ambitious TasP strategies are implemented, AF_early_ should thus increase even while incidence decreases. For instance, a recent phylodynamic analysis of Detroit’s population of men who have sex with men concluded that half of all transmission occurs within the first year of infection [49], and that individuals are 20 times as infectious in the first year post-infection (corresponding to EHM_acute_ = 228) [53]. However, this relative infectivity of the acute phase compares transmission from untreated, acutely infected individuals to that from treated, chronically infected individuals, and therefore overestimates relative acute phase infectivity. Future studies should interpret estimated AF_early_ in the context of ART coverage, noting that successful TasP interventions should increase AF_early_.

Finally, we again note arguments that because larger estimates of AF_early_ imply smaller reproductive numbers, TasP effectiveness may be less sensitive to AF_early_ in the long term than commonly assumed [4,13,15]. An observed epidemic trajectory can be explained by, at one extreme, infected individuals transmitting to relatively few people relatively quickly (low *R*_0_ but high AF_early_) or, at the other extreme, by infected individuals transmitting to relatively many people over a longer duration (high *R*_0_ but low AF_early_). Infectious diseases with smaller *R*_0_ are more sensitive to interventions [14]. Thus, in the former scenario, TasP would be proportionally less effective because AF_early_ is high, but reducing transmission would be easier (because *R*_0_ is low). In the latter scenario, TasP would avert a greater proportion of transmission, but population-level transmission would be more difficult to reduce (because *R*_0_ is high) [4,13]. Thus, the net effect of AF_early_ on the projected effectiveness of TasP interventions may be small [15], though this is still under debate [16]. Our results help to mediate the controversy over the impact of AF_early_ on intervention effectiveness. If AF_early_ is smaller than previously assumed, then any potential interference with TasP is also smaller and efforts to target early transmission may be less cost-effective, compared to more broad-scale interventions.

### Assumptions and Limitations

Biases arise when assumptions influence results but do not hold in the real world. The two prior studies that estimated HIV acute phase infectivity from the Rakai cohort data unknowingly suffered from four distinct sources of bias, each stemming from a specific problematic assumption. In our analysis, we used a detailed simulation model to explicitly correct these assumptions (S5 Table). While our model necessarily makes other assumptions, we have demonstrated that differences between our results and earlier analyses rest entirely on the four corrected assumptions. When making additional assumptions, we used the best available data, including age-at-seroconversion-dependent Weibull survival times [34]. Censorship rates due to loss to follow-up and couple dissolution [17,35,36] were informed by a recent study of the Rakai couples cohort [35] that largely overlapped with the original retrospective cohort study. While the exact number of incident SDCs excluded remains unknown, uncertainty in this quantity is reflected in our new estimates of EHM_acute_.

As with the preceding studies of acute phase infectivity [17,18], our goal was to accurately estimate the excess physiological infectivity due to the acute phase, which we measure by EHM_acute_. SDC cohort data that track susceptible individuals with both acutely infected and chronically infected partners is uniquely suited for this analysis. However, factors other than elevated physiological infectivity can also cause transmission in stable couples to occur more quickly from newly infected partners. In particular, various types of heterogeneity in infectiousness and susceptibility can lead to increased early transmission in couples cohorts and, if left unmodeled, can spuriously inflate estimates of physiologically elevated acute phase infectivity. While controlling for these confounding factors is critical, they should not all be dismissed as analytic nuisances. Some, but not all, forms of risk heterogeneity that bias couples cohort data can also transiently increase infectivity following infection in the broader population, and should be considered in addition to physiological infectivity when estimating AF_acute_ and designing interventions.

In particular, we distinguish between persistent heterogeneity and time-varying heterogeneity. Persistent heterogeneity arises from systematic variation between individuals in susceptibility or infectiousness that remains relatively stable over the course of individuals’ sexually active lifetimes. This includes persistent biological states (e.g., circumcision, host or virus genotypes, chronic co-infections) or persistent behavioral differences between individuals (e.g., condom usage). Highly susceptible individuals (or partners of highly effective transmitters) will be infected relatively quickly after their first exposure. In these cases, early transmission is not a consequence of high acute phase infectivity but instead of persistently high risk. Outside of the stable couple context, this mechanism will not create the same bias toward early transmission, because relationship initiation and fast transmission can happen during either the acute or the (longer) chronic phase.

The effects of time-varying heterogeneity are more complex. In some cases, time-varying heterogeneity in infectiousness can contribute to AF_early_ at the population level as strongly as in stable couples cohorts. This will occur when increased infectiousness is correlated with recency of infection. For example, newly infected individuals often have other sexually transmitted infections (STIs) that elevate HIV infectiousness (either because the STI increased their risk of acquiring HIV or because they acquired the STI and HIV through the same risk behaviors), and consequently are more infectious early after HIV infection prior to STI treatment [54]. This could, for instance, account for the observation in the Rakai cohort that, because incident couples exhibited a higher prevalence of GUD compared to prevalent couples, adjusting for self-reported GUD reduced estimated EHM_acute_. Unlike persistent heterogeneity, this mechanism would increase infectivity during the early phase not only within stable partnerships but also for the broader population. Temporal variation in safe sex practices or coital rate could cause similar effects. Time-varying heterogeneity was not incorporated because data were lacking regarding its magnitude and volatility and because, as discussed above, we expected it to contribute to early transmission in couples cohorts in a manner similar to that of persistent heterogeneity. Nonetheless, we did simulate persistent heterogeneity to explore how unmodeled heterogeneity biases EHM_acute_. Finally, some forms of risk heterogeneity will affect AF_early_ at the population level but cannot be observed in stable couples cohorts, because they do not affect transmission to a stable partner. Examples include partner acquisition rates and tendency to maintain concurrent relationships (i.e., episodic risk behavior [11,12]).

**Fig 7:**
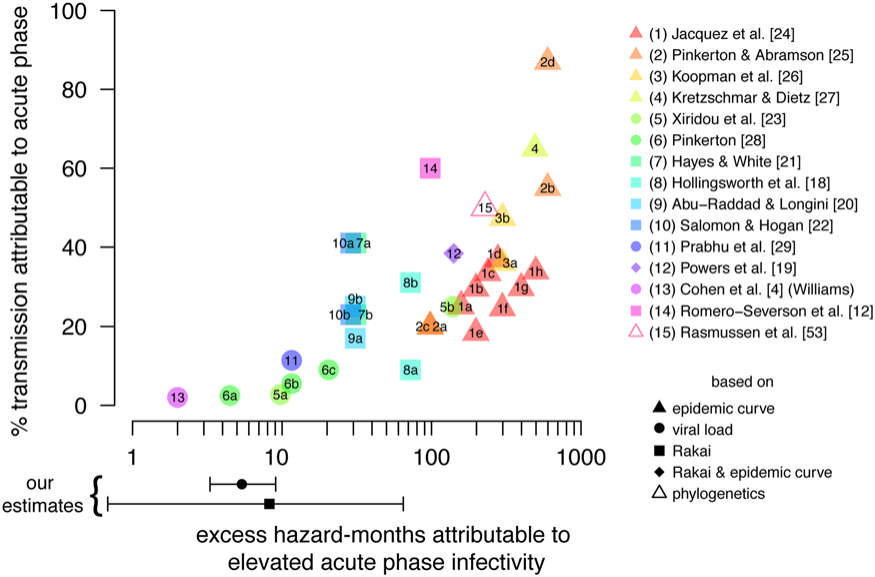
Proportion of transmission due to acute infectivity. Published estimates of the proportion of incidence attributable to early transmission (AF_early_) versus the assumed excess hazard-months attributable to physiologically elevated acute phase infectivity (EHM_acute_). Shapes indicate whether EHM_acute_ was estimated from epidemic growth rates, viral load trajectories and viral load–infectivity relationships, the Rakai retrospective cohort, phylogenetics, or a combination thereof. Points reflecting studies that published more than one result are identified with letters; explanations of differences between estimates are available in S6 Table. Points and error bars below the *x*-axis indicate our estimated EHM_acute_ from the Rakai retrospective cohort data and based on viral load trajectories; we do not specify a sexual network model and therefore do not estimate AF_early_ in this study.

While we separate physiologically elevated acute phase infectivity from various types of heterogeneity, we emphasize that estimates of AF_early_ must consider not only EHM_acute_ and sexual network assumptions, but also sources of heterogeneity that could potentially amplify early transmission at the population level, including some that have been observed in the couples cohort that we have separated from EHM_acute_. Thus, while studies assuming larger values of EHM_acute_ have generally produced larger estimates of AF_early_ (Fig. 7; S6 Table), we suggest caution when considering the intuitive conclusion that studies relying on upward-biased estimates of EHM_acute_ have also overestimated AF_early_. Because our analysis focused on estimating the relative infectivity and duration of the acute phase from SDCs, our model did not specify population-level sexual mixing patterns and thus was unable to produce an estimate of AF_early_. We believe new estimates of AF_early_ are needed that carefully consider our updated EHM_acute_ estimate, along with both persistent and time-varying heterogeneity, sexual network assumptions, and explicit consideration of differences in ART coverage between acutely and chronically infected individuals.

## Conclusion

By analyzing a seminal HIV couples cohort study using stochastic models and approximate Bayesian computation, we have reestimated the relative infectivity of the acute phase and found that the most highly cited estimates are substantially biased upward by unmodeled heterogeneity and by the study exclusion criteria. Thus, the proportion of transmission occurring immediately after infection should be reevaluated, and may have more to do with risk heterogeneity and HIV intervention measures than with physiological differences between the acute and chronic stages of infection. These revised estimates should be considered when designing population-scale interventions and communicating individual-level risk in clinical or community settings. It is becoming increasingly clear that infected individuals should initiate ART as early as possible both to achieve the greatest reductions in transmission and for its direct clinical benefits [55]. Our findings cautiously suggest that the population-level benefits might be larger than predicted by earlier estimates.

## Supporting Information

S1 Data New best estimates of acute phase infectivity. This ZIP file contains an RDATA file intended to facilitate future modeling work by providing our ABC-SMC posterior distribution for parameter estimates for acute phase infectivity and duration, and individual heterogeneity in transmission.
(ZIP)

S1 Fig Fit of model to the Rakai retrospective cohort. Left column shows the proportion of secondary partners seroconverting in incident couples for each of the four intervals of observation from our posterior (black) and from the Rakai data (red). Right column shows the same for the prevalent couples.
(TIF)

S2 Fig True EHM_acute_ for various simulation and analysis scenarios. This figure is analogous to the results in Fig. 3 but shows the actual point estimates and 95% confidence (Poisson regression model) or crediblility (variable hazard survival model) intervals for model fits to simulations. Lines in Fig. 6 show the loess fits to these trends, with the same lines shown here (line types and colors match Fig. 6). The top row shows estimates acquired using the unadjusted Poisson regression (Fig. 6B) [17]. The middle row shows estimates from a Poisson regression in which 50% of heterogeneity is controlled for (Fig. 6D; here we show 50% for different σ_hazard_ values instead of 25%, 50%, and 80% for just σ_hazard_ = 3). The bottom row shows estimates acquired by fitting the variable hazard survival model [18] to the data (Fig. 6C). The leftmost column shows analyses from retrospective cohorts without heterogeneity, with all incident SDCs included regardless of follow-up, and with no elevated late phase infectivity. Columns 2–6 show analyses of simulations that include elevated late phase infectivity. Columns 3–6 show analyses that exclude incident SDCs observed only once and then lost to follow-up (Fig. 4). Columns 4–6 display analyses of simulations with increasing amounts of heterogeneity (as measured by the standard deviation of the log-hazard, σ_hazard_).
(TIF)

S3 Fig Biases caused by the assumption that events occur at interval midpoints. The left panel shows the average person-months at risk in secondary partners in prevalent SDCs stratified by their infection status at the end of the interval (orange = infected, red = uninfected) as well as the average amongst all couples (black). The dashed lines show the midpoint interval assumptions; there was an assumed 10 mo of person-months of risk for partners who remained uninfected throughout the interval and an assumed 5 mo for those who became infected. The arrow shows the estimated hazard of transmission within SDCs in Rakai. While the midpoint assumption holds well in prevalent couples, the right panel shows that the assumptions do not hold as well for incident couples, in which it’s assumed that the first partner was infected at 5 mo and the second partner is then exposed to this infectious partner for 2.5 mo if they get infected and 5 mo if they do not. In fact, the person-time exposed is a function of the hazard itself, and the average is always less than 5 mo and sometimes less than 2.5 mo. This occurs because, for increasing hazards, secondary partner infections occur soon after index partner infections, and the only secondary partners who avoid being infected are those whose index partner was infected very late in the 10-mo interval, such that they experienced very little person-time of exposure.
(TIF)

S4 Fig Validation using data generated by the variable hazard survival model. Estimated versus true simulated EHM_acute_ when fitting the Hollingsworth et al. [18] variable hazard survival model to either data simulated by this model (dashed lines) or data simulated by the full couples transmission model used in the main text (solid lines). For the data-generating model based on the variable hazard survival model, we simulated incident, prevalent, and late SDCs as three categorically different groups (i.e., there could be no misclassification between groups); did not allow for loss to follow-up; and allowed the timing of an event (infection or death) within a 10-mo interval to be distributed with equal probability throughout that interval. Our own data-generating model relied on a simulated couples population, with a retrospective cohort identified afterwards. As expected, fitting the variable hazard survival model to data generated by the same model produced accurate results (i.e., compare with S3 Fig., third row, second column). When fitting to data from our more realistic couples model in the scenario where no seroincident couples were excluded and transmission was homogenous, estimates of EHM_acute_ from the variable hazard survival model were biased downward (i) because late SDCs were sometimes misclassified as prevalent SDCs when couples were loss to follow-up shortly before a partner died; this happened more frequently for greater excess hazard-months attributable to the late and AIDS phases (EHM_late_). This resulted in upward-biased estimates of the chronic phase hazard (i.e., chronic transmission is partly contaminated by late transmission) and, subsequently, biased the acute to chronic phase relative hazard (RH_acute_) and EHM_acute_ downward.
(TIF)

S5 Fig Proportion of incident couples excluded. (A) Proportion of incident SDCs excluded by Wawer et al. [17] exclusion criteria versus EHM_acute_ from our posterior ABC-SMC fitted parameters. The median proportion excluded was 43% (95% CI: 27%–60%). (B) Posterior distribution of the number of couples excluded calculated as 23/(1 − proportion excluded) − 23, where 23 is the number included in the study. The median number excluded was 17 (95% CI: 8–35). We specified that incident SDCs had a 47% probability of being lost to follow-up in the subsequent interval (red line). Variation in the number of couples excluded emerges both from stochastic variation in the combined loss-to-follow-up and couple dissolution process and from the number of couples who were censored and excluded because the first visit at which they were observed serodiscordant occurred during the last cohort visit of the study.
(TIF)

S6 Fig Effect of excluding couples in which the second partner was infected by extra-couple transmission. Solid lines replicate Fig. 6B and 6C, except that dashed lines in Fig. 6 are shown here as dark gray lines, and dotted lines in Fig. 6 are shown here as light gray lines. In this figure, dashed lines show analyses of simulated cohorts that, in contrast to the main analyses, included couples in which the second partner was infected by extra-couple transmission, with the couples censored starting from the interval during which the extra-couple infection occurred. The exclusion of these couples did not cause a systematic bias. This was because EHM_acute_ compares hazard between incident and prevalent couples. Since the person-time excluded in incident couples was balanced by that excluded in prevalent couples, the two effects approximately balanced each other.
(TIF)

S7 Fig Infectivity versus viral load from multiple studies. Fig. 2A shows Lingappa et al.’s fitted log-linear model of HIV transmission hazard by viral load [7]. Here, we show all available infectivity by viral load data to show that this trend is characteristic and, in fact, conservative. Attia et al.’s meta-analysis of all relevant data up to that point [6] suggests a more saturating curve, with increases in infectivity appearing to hit an asymptote at 10^4.5^ copies/ml. If this relationship saturates, then the acute phase infectivity would be expected to be even closer to that of the chronic phase based on viral load curves alone, and our estimated EHM_acute_ from viral load trajectories in the main text is conservatively high.
(TIF)

S8 Fig Survival-inflated copula model of couple relationship histories for Uganda. (A) Pairwise density plots of the five variables that comprise each couple’s relationship history from the Ugandan Demographic and Health Survey: age at male and female sexual debut (ams and afs), male and female duration of sexual activity prior to couple formation (mdur and fdur), and date of couple formation (tmar). All values are shown in months or months since 1900. (B) Our multivariate copula distribution model fit to these data, from which we simulated couples representative of the multivariate correlated relationship between these variables in Uganda. Note that we simulated the first four variables conditional on the last (tmar), where couples cohorts (defined by date of couple formation) of equal size were used for the period simulated.
(TIF)

S9 FigApproximate Bayesian computation fits to acute phase and hazard parameters.Figure shows the prior distribution, intermediate distributions, and final posterior distribution generated by an ABC-SMC fit of our couples transmission model to the Rakai retrospective cohort data. Parameters shown include (A) the acute to chronic phase relative hazard, RH_acute_; (B) the duration of the acute phase, *d*_acute_; (C) the mean monthly within-couple transmission rate, ${\dot{\lambda }}_{\text{hazard}}$(we give the median [${\bar{\lambda }}_{\text{hazard}}$] in the main text, since the mean is in the upper tail of the log-normal distribution); and (D) the standard deviation of the risk distribution governing the amount of individual heterogeneity, σ_hazard_. In (A–C), *x*-axes are shown on the log scale. The convergence of sequential intermediate distributions from each sequential Monte Carlo step suggests that the fifth iteration is an adequate representation of the posterior.(TIF)

S1 Table Prior distributions used for ABC-SMC fitting procedure. Parameters are shown on the transformation over which they were sampled (i.e., logarithm or not). All priors were uninformative uniform distributions except for the ratios between male and female transmission coefficients for each transmission route (ρ_b_, ρ_e_, ρ*), which were log-normal distributions based on posterior estimates of these parameters from our fit of this couples transmission model to Demographic and Health Survey data in Uganda.
(DOCX)

S2 TableSummary statistics and threshold criteria for ABC-SMC.For each of *t* = 1,...,5 sequential Monte Carlo iterations, we applied the following criteria to determine whether a parameter particle θ** was included in the intermediate distribution {$\theta_{t}^{\left(i\right)}$}. See Section VII of S1 Text for a detailed explanation of each summary statistic.(DOCX)

S3 Table Parameter ranges simulated. We generated couples cohort simulations over the entire range of parameters specified in this table. The chronic phase was defined as the period of time after the acute phase and before the late phase, and varied in duration depending on an individual’s survival time (i.e., fast progressors had shorter chronic phases).
(DOCX)

S4 Table Data from Table 1 in Wawer et al. [17]. Rows in gray indicate observation intervals that were excluded from the Wawer et al. Poisson regression analysis because they were not believed to be reflective of the phase of interest (i.e., acute and late for incident and late couples, respectively). Data from all intervals were, in contrast, used by Hollingsworth et al.’s fit of a variable hazard survival model [18].
(DOCX)

S5 Table Assumptions made by previous analyses of the Rakai retrospective cohort that are relaxed in our reanalysis. (DOCX)

S6 Table Summary of *d*_acute_, RH_acute_, and resulting EHM_acute_ values used in studies aiming to estimate AF_acute_. Fig. 7 in the main text plots AF_acute_ versus EHM_acute_ for these studies; superscripted numbers and letters refer to the legend in Fig. 7. We do not provide confidence intervals on estimates because most studies that estimated precision used sensitivity analyses with qualitatively different justifications.
(DOCX)

S1 Text Online supplementary appendix. (DOCX)

## Abbreviations

95% CI: 95% confidence interval
95% CrI: 95% credibility interval
ABC-SMC: approximate Bayesian computation with sequential Monte Carlo
AF_acute_: fraction of HIV incidence attributable to transmission from acutely infected individuals
AF_early_: fraction of HIV incidence attributable to transmission early after infection
ART: antiretroviral therapy; dacute, acute phase duration
EHM_acute_: excess hazard-months attributable to the acute phase
GUD: genital ulcer disease
RH_acute_: relative hazard of transmission during the acute versus chronic phase
SDC: serodiscordant couple
STI: sexually transmitted infection
TasP: treatment as prevention
