# Supplementary figures and images for: Reassessment of HIV-1 Acute Phase Infectivity: Accounting for Heterogeneity and Study Design with Simulated Cohorts
Source: PLoS Med. 2015 Mar 17;12(3):e1001801. doi: 10.1371/journal.pmed.1001801 (PMC4363602; doi:10.1371/journal.pmed.1001801)

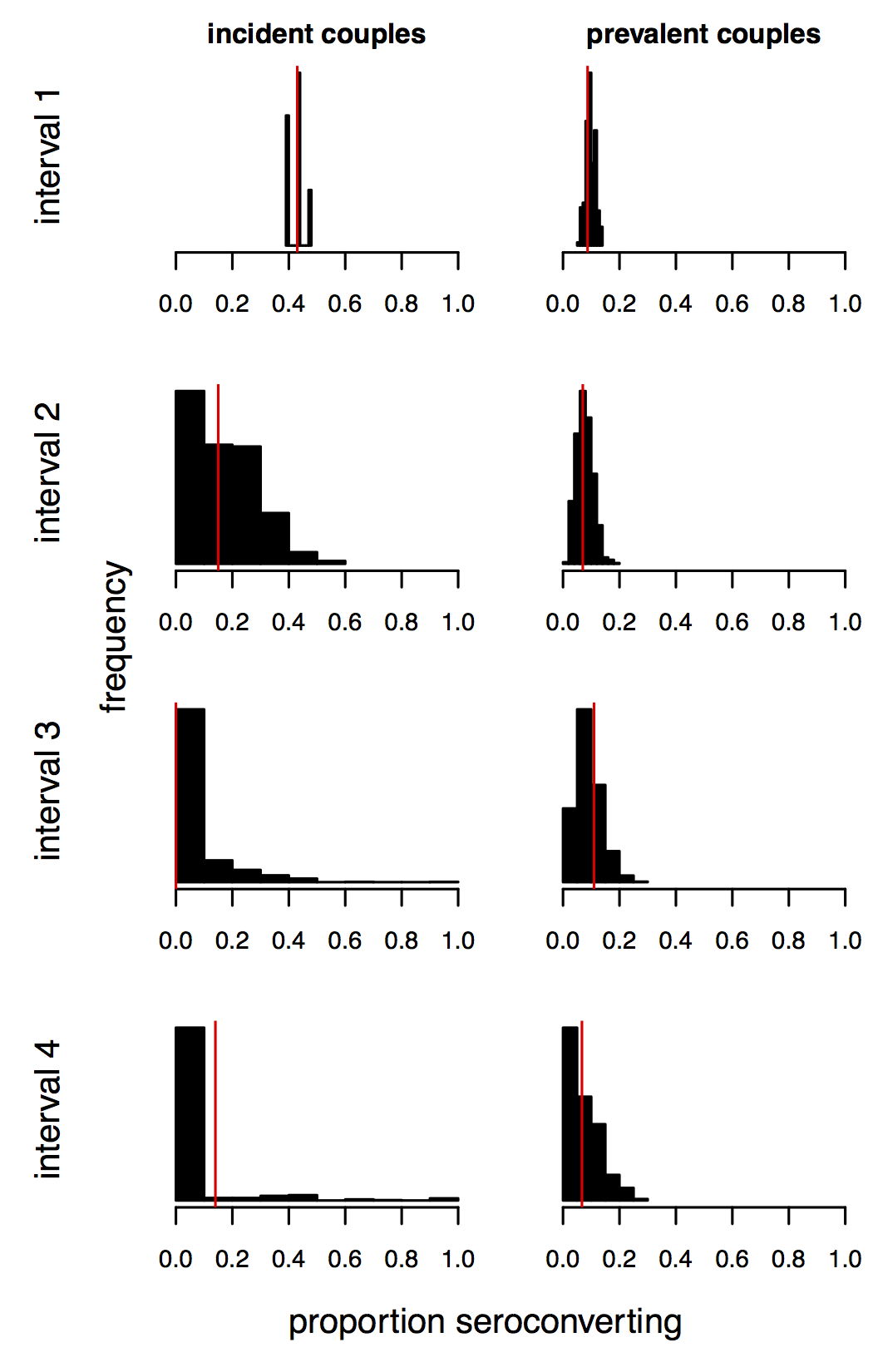

Supplement: S1 Fig — Left column shows the proportion of secondary partners seroconverting in incident couples for each of the four intervals of observation from our posterior (black) and from the Rakai data (red). Right column shows the same for the prevalent couples. (TIF) [file pmed.1001801.s002.tif]

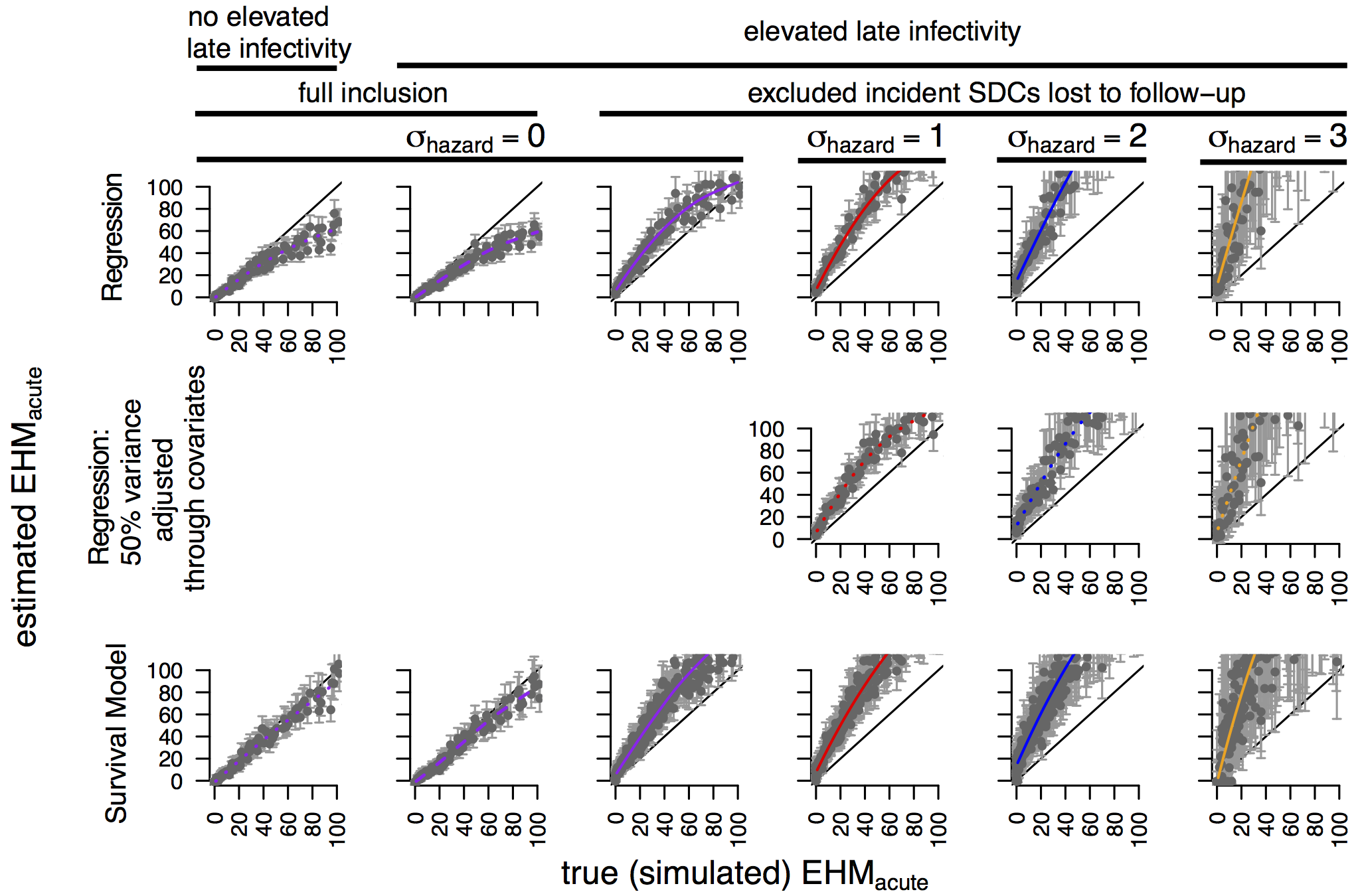

Supplement: S2 Fig — This figure is analogous to the results in Fig. 3 but shows the actual point estimates and 95% confidence (Poisson regression model) or crediblility (variable hazard survival model) intervals for model fits to simulations. Lines in Fig. 6 show the loess fits to these trends, with the same lines shown here (line types and colors match Fig. 6). The top row shows estimates acquired using the unadjusted Poisson regression (Fig. 6B) [17]. The middle row shows estimates from a Poisson regression in which 50% of heterogeneity is controlled for (Fig. 6D; here we show 50% for different σhazard values instead of 25%, 50%, and 80% for just σhazard = 3). The bottom row shows estimates acquired by fitting the variable hazard survival model [18] to the data (Fig. 6C). The leftmost column shows analyses from retrospective cohorts without heterogeneity, with all incident SDCs included regardless of follow-up, and with no elevated late phase infectivity. Columns 2–6 show analyses of simulations that include elevated late phase infectivity. Columns 3–6 show analyses that exclude incident SDCs observed only once and then lost to follow-up (Fig. 4). Columns 4–6 display analyses of simulations with increasing amounts of heterogeneity (as measured by the standard deviation of the log-hazard, σhazard). (TIF) [file pmed.1001801.s003.tif]

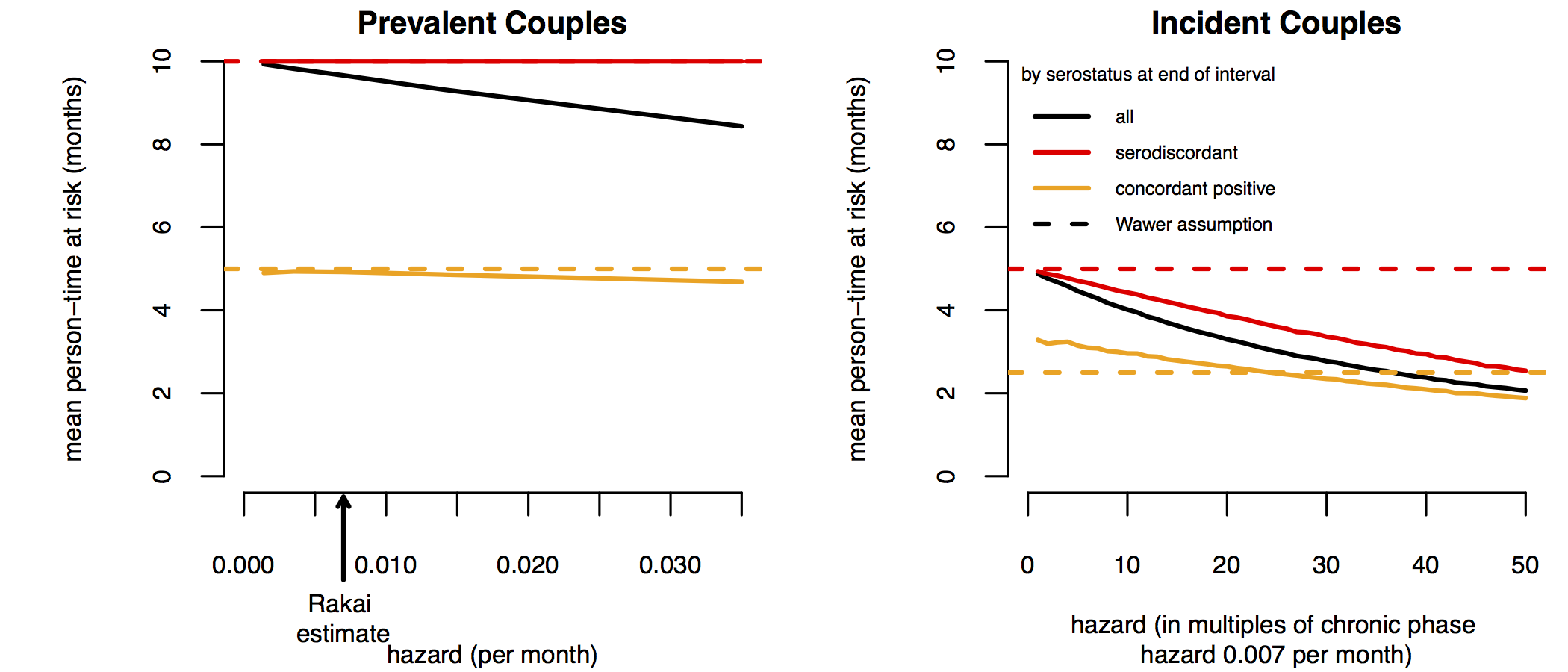

Supplement: S3 Fig — The left panel shows the average person-months at risk in secondary partners in prevalent SDCs stratified by their infection status at the end of the interval (orange = infected, red = uninfected) as well as the average amongst all couples (black). The dashed lines show the midpoint interval assumptions; there was an assumed 10 mo of person-months of risk for partners who remained uninfected throughout the interval and an assumed 5 mo for those who became infected. The arrow shows the estimated hazard of transmission within SDCs in Rakai. While the midpoint assumption holds well in prevalent couples, the right panel shows that the assumptions do not hold as well for incident couples, in which it’s assumed that the first partner was infected at 5 mo and the second partner is then exposed to this infectious partner for 2.5 mo if they get infected and 5 mo if they do not. In fact, the person-time exposed is a function of the hazard itself, and the average is always less than 5 mo and sometimes less than 2.5 mo. This occurs because, for increasing hazards, secondary partner infections occur soon after index partner infections, and the only secondary partners who avoid being infected are those whose index partner was infected very late in the 10-mo interval, such that they experienced very little person-time of exposure. (TIF) [file pmed.1001801.s004.tif]

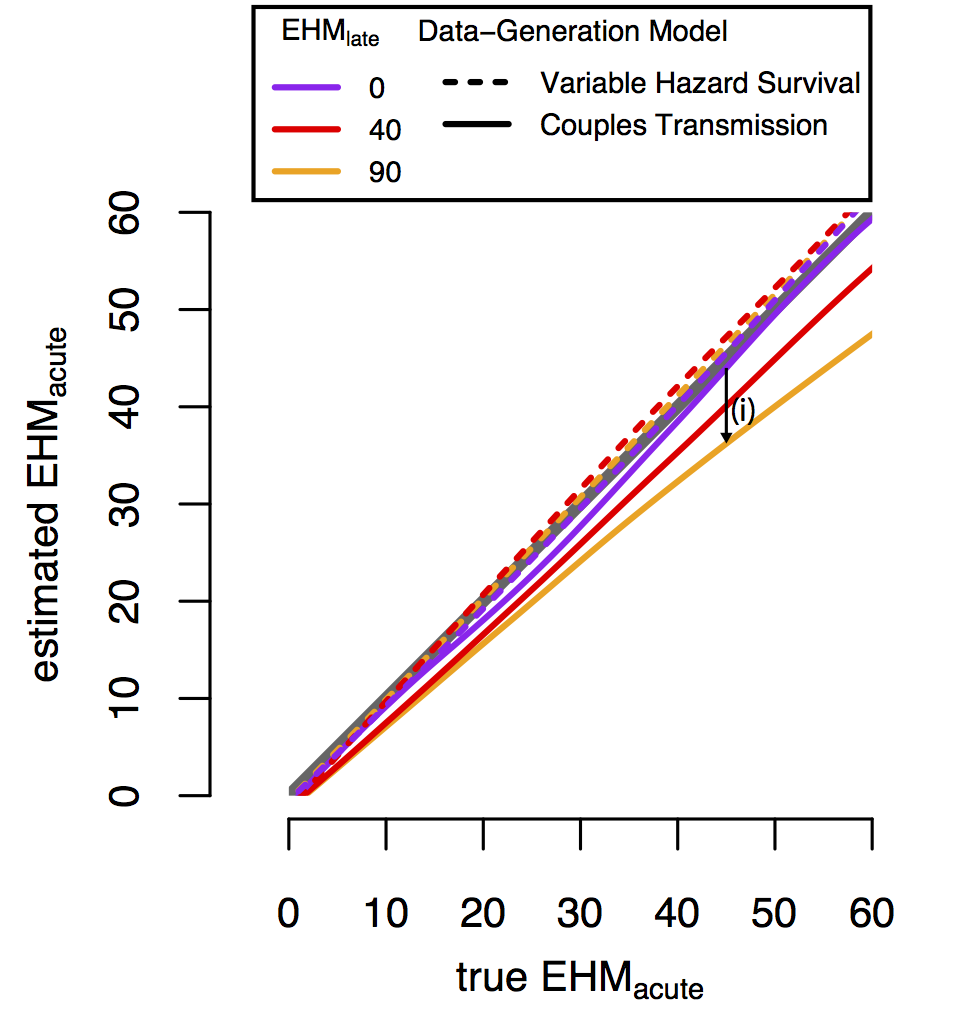

Supplement: S4 Fig — Estimated versus true simulated EHMacute when fitting the Hollingsworth et al. [18] variable hazard survival model to either data simulated by this model (dashed lines) or data simulated by the full couples transmission model used in the main text (solid lines). For the data-generating model based on the variable hazard survival model, we simulated incident, prevalent, and late SDCs as three categorically different groups (i.e., there could be no misclassification between groups); did not allow for loss to follow-up; and allowed the timing of an event (infection or death) within a 10-mo interval to be distributed with equal probability throughout that interval. Our own data-generating model relied on a simulated couples population, with a retrospective cohort identified afterwards. As expected, fitting the variable hazard survival model to data generated by the same model produced accurate results (i.e., compare with S3 Fig., third row, second column). When fitting to data from our more realistic couples model in the scenario where no seroincident couples were excluded and transmission was homogenous, estimates of EHMacute from the variable hazard survival model were biased downward (i) because late SDCs were sometimes misclassified as prevalent SDCs when couples were loss to follow-up shortly before a partner died; this happened more frequently for greater excess hazard-months attributable to the late and AIDS phases (EHMlate). This resulted in upward-biased estimates of the chronic phase hazard (i.e., chronic transmission is partly contaminated by late transmission) and, subsequently, biased the acute to chronic phase relative hazard (RHacute) and EHMacute downward. (TIF) [file pmed.1001801.s005.tif]

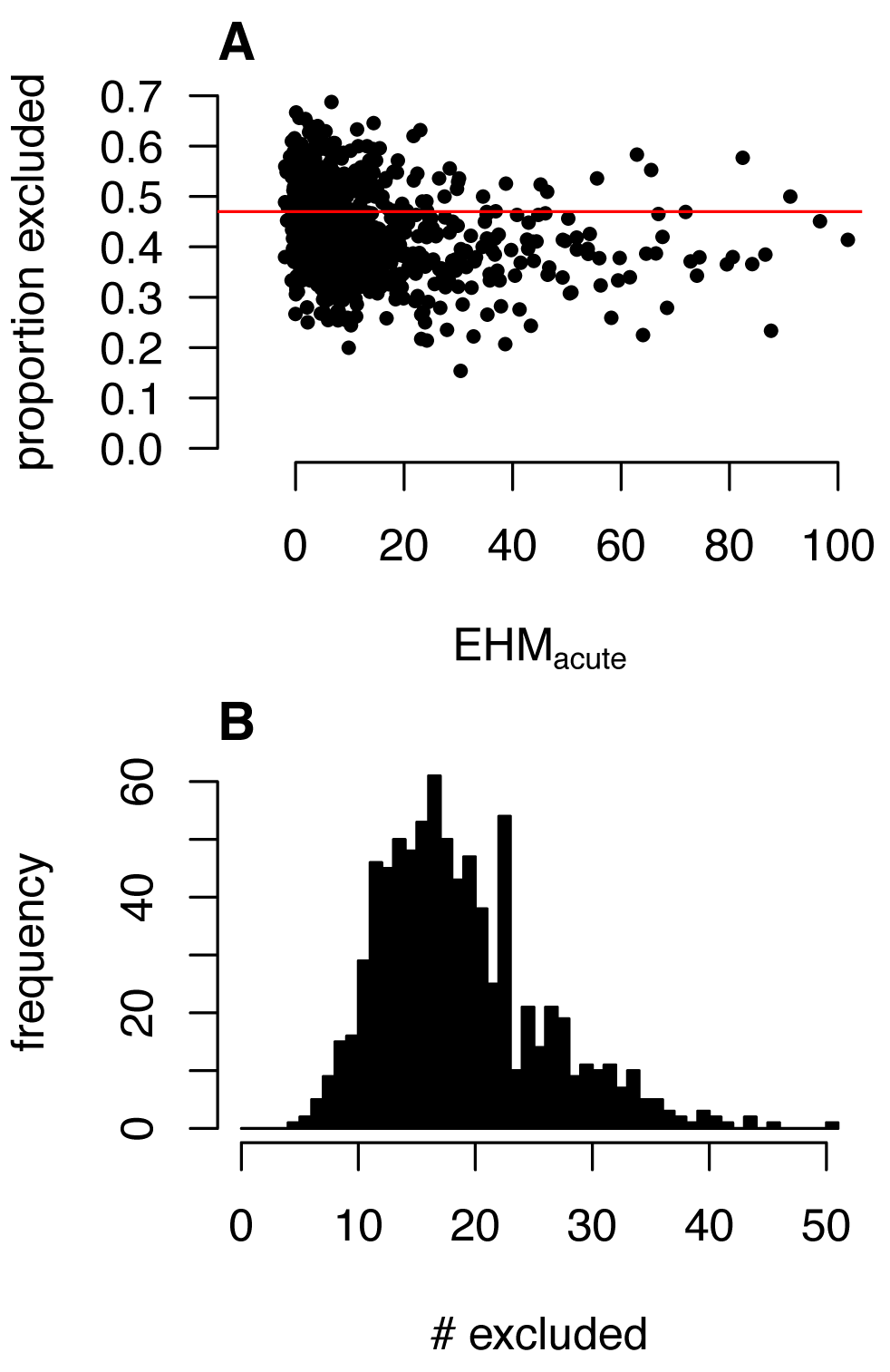

Supplement: S5 Fig — (A) Proportion of incident SDCs excluded by Wawer et al. [17] exclusion criteria versus EHMacute from our posterior ABC-SMC fitted parameters. The median proportion excluded was 43% (95% CI: 27%–60%). (B) Posterior distribution of the number of couples excluded calculated as 23/(1 − proportion excluded) − 23, where 23 is the number included in the study. The median number excluded was 17 (95% CI: 8–35). We specified that incident SDCs had a 47% probability of being lost to follow-up in the subsequent interval (red line). Variation in the number of couples excluded emerges both from stochastic variation in the combined loss-to-follow-up and couple dissolution process and from the number of couples who were censored and excluded because the first visit at which they were observed serodiscordant occurred during the last cohort visit of the study. (TIF) [file pmed.1001801.s006.tif]

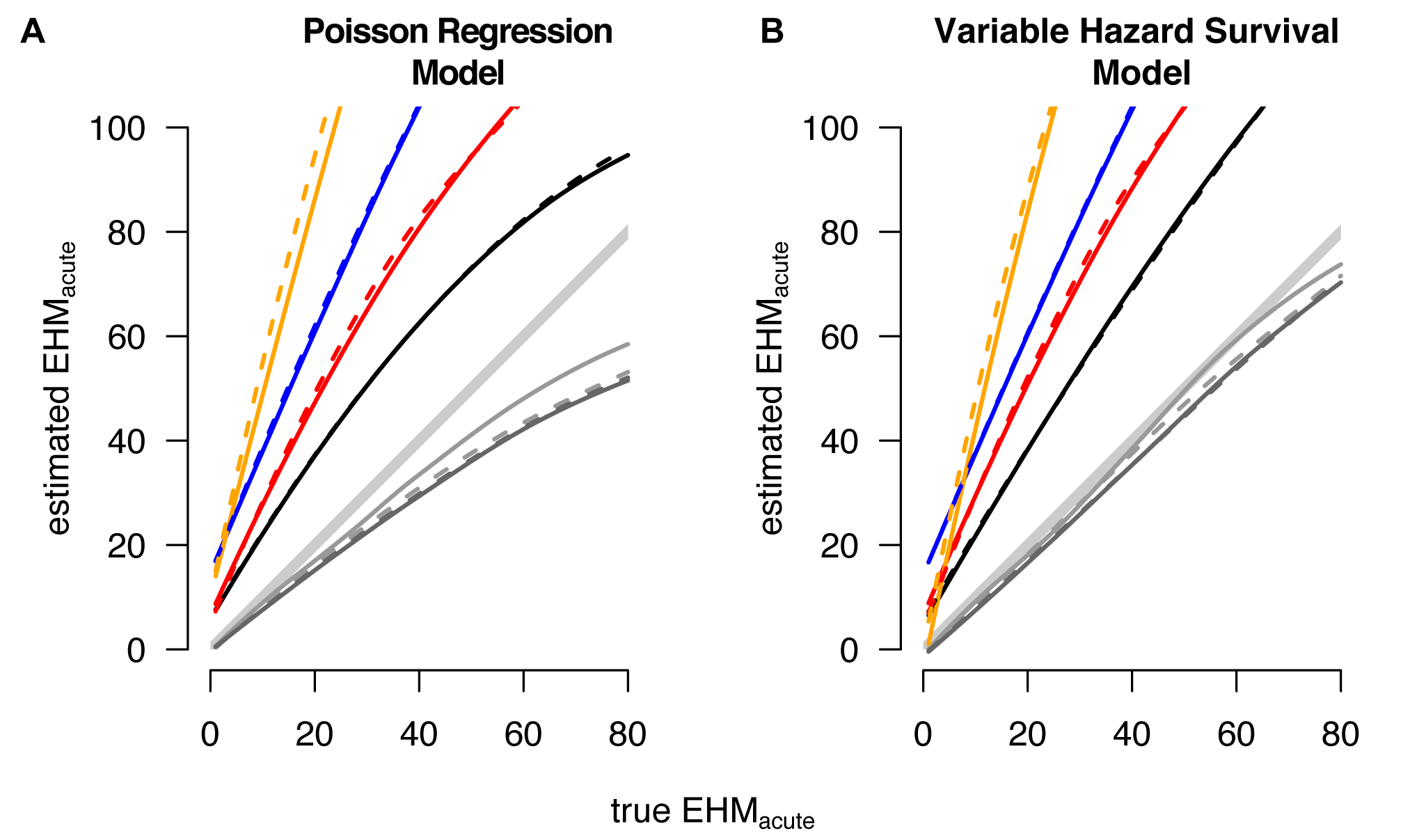

Supplement: S6 Fig — Solid lines replicate Fig. 6B and 6C, except that dashed lines in Fig. 6 are shown here as dark gray lines, and dotted lines in Fig. 6 are shown here as light gray lines. In this figure, dashed lines show analyses of simulated cohorts that, in contrast to the main analyses, included couples in which the second partner was infected by extra-couple transmission, with the couples censored starting from the interval during which the extra-couple infection occurred. The exclusion of these couples did not cause a systematic bias. This was because EHMacute compares hazard between incident and prevalent couples. Since the person-time excluded in incident couples was balanced by that excluded in prevalent couples, the two effects approximately balanced each other. (TIF) [file pmed.1001801.s007.tif]

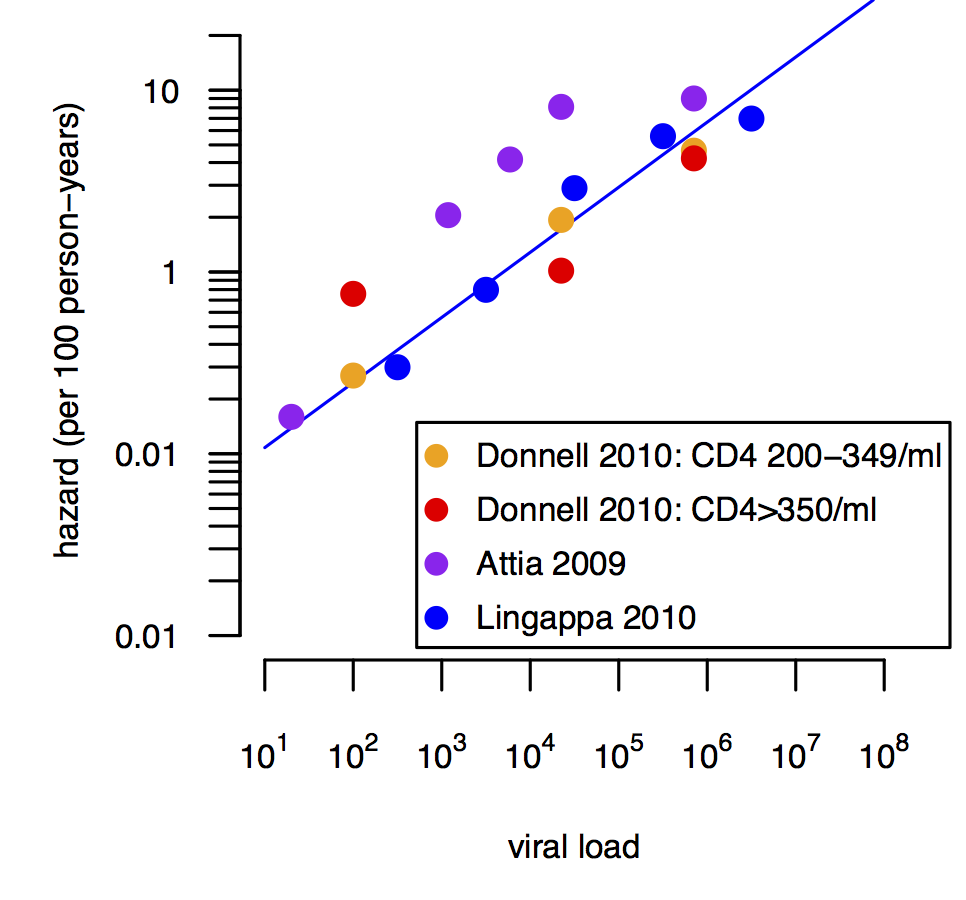

Supplement: S7 Fig — Fig. 2A shows Lingappa et al.’s fitted log-linear model of HIV transmission hazard by viral load [7]. Here, we show all available infectivity by viral load data to show that this trend is characteristic and, in fact, conservative. Attia et al.’s meta-analysis of all relevant data up to that point [6] suggests a more saturating curve, with increases in infectivity appearing to hit an asymptote at 104.5 copies/ml. If this relationship saturates, then the acute phase infectivity would be expected to be even closer to that of the chronic phase based on viral load curves alone, and our estimated EHMacute from viral load trajectories in the main text is conservatively high. (TIF) [file pmed.1001801.s008.tif]

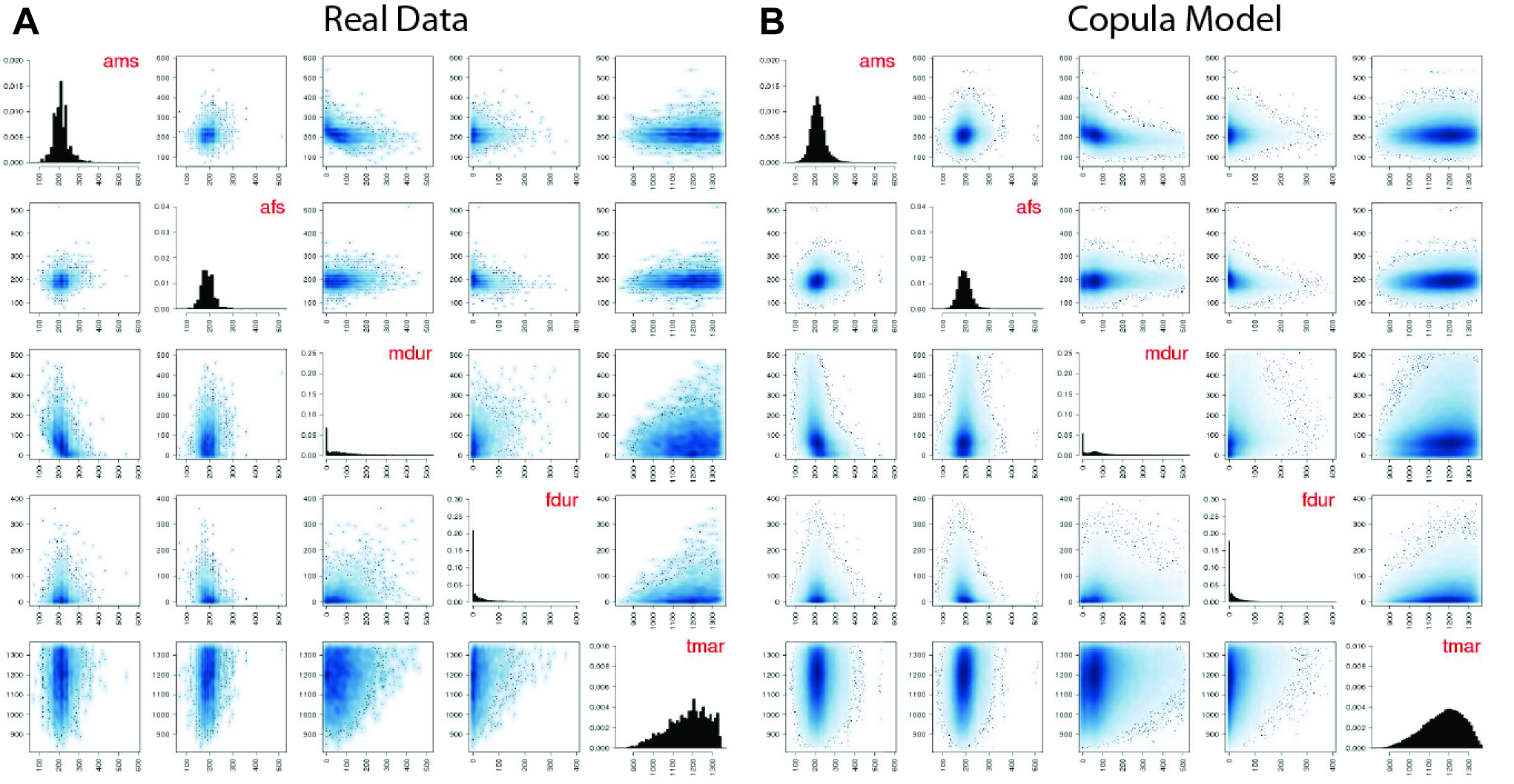

Supplement: S8 Fig — (A) Pairwise density plots of the five variables that comprise each couple’s relationship history from the Ugandan Demographic and Health Survey: age at male and female sexual debut (ams and afs), male and female duration of sexual activity prior to couple formation (mdur and fdur), and date of couple formation (tmar). All values are shown in months or months since 1900. (B) Our multivariate copula distribution model fit to these data, from which we simulated couples representative of the multivariate correlated relationship between these variables in Uganda. Note that we simulated the first four variables conditional on the last (tmar), where couples cohorts (defined by date of couple formation) of equal size were used for the period simulated. (TIF) [file pmed.1001801.s009.tif]

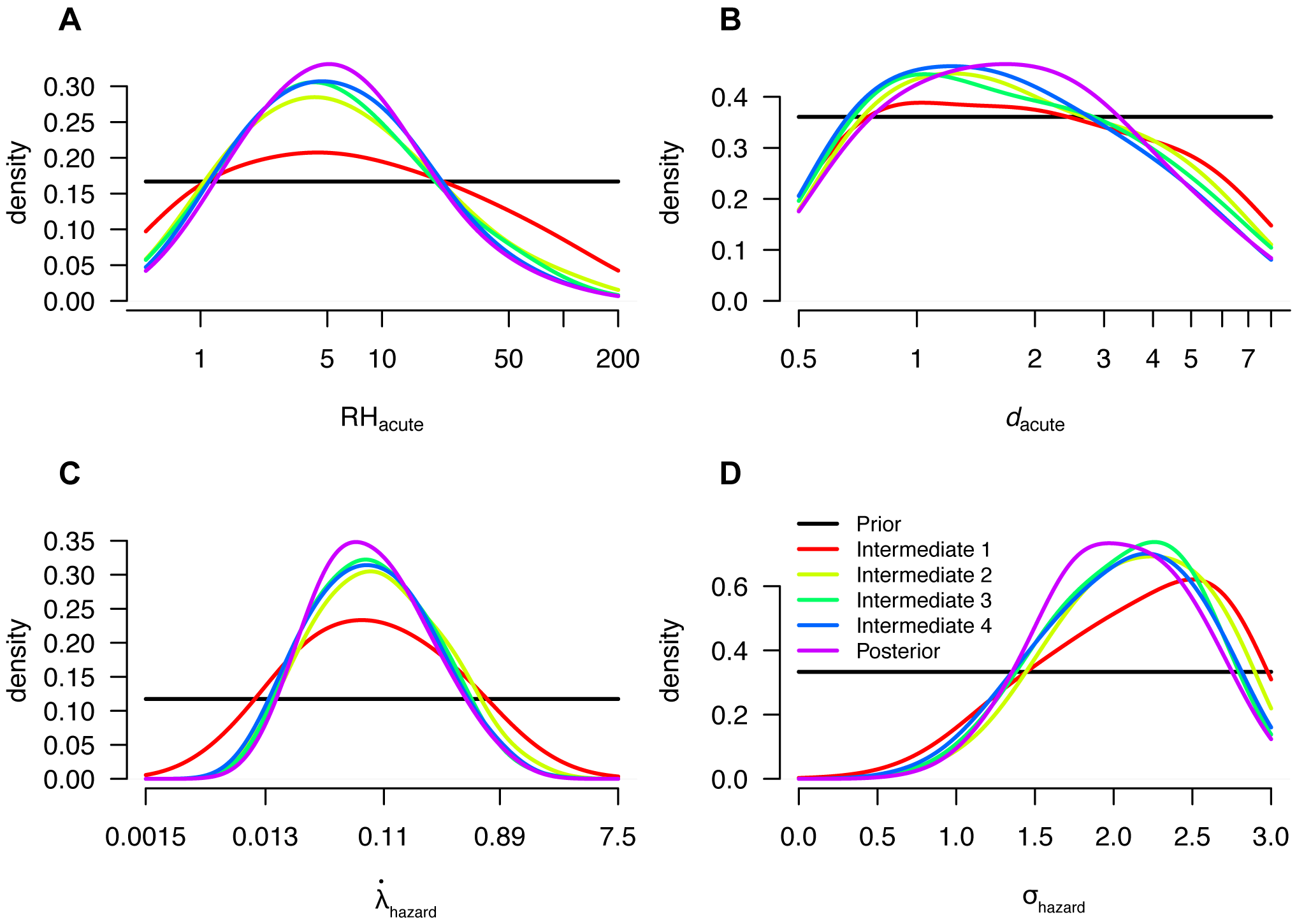

Supplement: S9 Fig — Figure shows the prior distribution, intermediate distributions, and final posterior distribution generated by an ABC-SMC fit of our couples transmission model to the Rakai retrospective cohort data. Parameters shown include (A) the acute to chronic phase relative hazard, RHacute; (B) the duration of the acute phase, d acute; (C) the mean monthly within-couple transmission rate, λ˙hazard(we give the median [λ¯hazard] in the main text, since the mean is in the upper tail of the log-normal distribution); and (D) the standard deviation of the risk distribution governing the amount of individual heterogeneity, σhazard. In (A–C), x-axes are shown on the log scale. The convergence of sequential intermediate distributions from each sequential Monte Carlo step suggests that the fifth iteration is an adequate representation of the posterior. (TIF) [file pmed.1001801.s010.tif]
