# Supplementary material for: Reassessment of HIV-1 Acute Phase Infectivity: Accounting for Heterogeneity and Study Design with Simulated Cohorts
Source: PLoS Med. 2015 Mar 17;12(3):e1001801. doi: 10.1371/journal.pmed.1001801 (PMC4363602; doi:10.1371/journal.pmed.1001801)
Supplement: S1 Table — Parameters are shown on the transformation over which they were sampled (i.e., logarithm or not). All priors were uninformative uniform distributions except for the ratios between male and female transmission coefficients for each transmission route (ρb, ρe, ρ*), which were log-normal distributions based on posterior estimates of these parameters from our fit of this couples transmission model to Demographic and Health Survey data in Uganda. (DOCX) [file pmed.1001801.s011.docx]

S1 Table. Prior distributions used for ABC-SMC fitting procedure. Parameters are shown on the transformation over which they were sampled (i.e. logarithm or not). All priors were uninformative uniform distributions except for the ratios between male and female transmission coefficients for each transmission route ($\boldsymbol{\rho}_{\mathbf{b}}\boldsymbol{,}\boldsymbol{\rho}_{\mathbf{e}}\boldsymbol{,}\boldsymbol{\rho}^{\boldsymbol{*}}$), which were lognormal distributions based on posterior estimates of these parameters from our fit of this couple transmission model to DHS data in Uganda. Note that we use $\bar{\beta}^{*}$ and $\dot{\lambda}_{\mathrm{hazard}}$ interchangeably in the main text as the geometric mean across sexes expected value of the chronic phase transmission rate.

| Parameter | Prior | Parameter | Prior | Parameter | Prior |
| --- | --- | --- | --- | --- | --- |
| $\mathbf{log}\boldsymbol{(}{\bar{\boldsymbol{\beta}}}_{\mathbf{b}}\boldsymbol{)}$ | unif(-5.87, -1.96) | $log(\rho_{b})$ | $\mathcal{N}$(-0.853, 0.234) | log(RH_acute_) | unif(log(0.5), log(200)) |
| $\mathbf{log}\boldsymbol{(}{\bar{\boldsymbol{\beta}}}_{\mathbf{e}}\boldsymbol{)}$ | unif(-7.09, -3.18) | $log(\rho_{e})$ | $\mathcal{N}$(-0.214, 0.289) | log(d_acute_) | unif(log(0.5), log(8)) |
| $\mathbf{log}\boldsymbol{(}{\bar{\boldsymbol{\beta}}}^{\boldsymbol{*}}\boldsymbol{)}$ | unif(-6.50, 2.02) | $log(\rho^{*})$ | $\mathcal{N}$(-0.399, 0.364) | $\sigma_{\mathrm{hazard}}$ | unif(0, 3) |
