# Supplementary material for: Reassessment of HIV-1 Acute Phase Infectivity: Accounting for Heterogeneity and Study Design with Simulated Cohorts
Source: PLoS Med. 2015 Mar 17;12(3):e1001801. doi: 10.1371/journal.pmed.1001801 (PMC4363602; doi:10.1371/journal.pmed.1001801)
Supplement: S2 Table — For each of t = 1,...,5 sequential Monte Carlo iterations, we applied the following criteria to determine whether a parameter particle θ** was included in the intermediate distribution {θt(i)}. See Section VII of S1 Text for a detailed explanation of each summary statistic. (DOCX) [file pmed.1001801.s012.docx]

S2 Table. Summary statistics and threshold criteria for ABC-SMC. For each of $\boldsymbol{t=}\boldsymbol{1,\ldots,5}$ sequential Monte Carlo iterations we applied the following criteria to determine whether a parameter particle $\boldsymbol{\theta}^{\boldsymbol{**}}$ was included in the intermediate distribution {$\boldsymbol{\theta}_{\boldsymbol{t}}^{\left( \boldsymbol{i} \right)}\boldsymbol{\}}$. See Section VII of S1 Text for a detailed explanation of each summary statistic.

| Summary Statistic | Threshold Criteria for SMC iteration *t* | Justification |
| --- | --- | --- |
| $\boldsymbol{N}_{\mathbf{inc}}^{\boldsymbol{*}}$ | $N_{inc,Min,t}<N_{\mathrm{inc}}^{*}<N_{inc,Max,t}$   \| $\boldsymbol{t}$ \| 1 \| 2 \| 3 \| 4 \| 5 \| \| --- \| --- \| --- \| --- \| --- \| --- \| \| $\boldsymbol{N}_{\mathbf{inc,Min,t}}$ \| 20 \| 23 \| 23 \| 23 \| 23 \| \| $\boldsymbol{N}_{\mathbf{inc,Max,t}}$ \| 80 \| 60 \| 60 \| 60 \| 60 \| | Ensure that transmission rates into couples and between partners generate a similar number of incident couples to the real data size given a couple cohort of size 4,875. |
| $\boldsymbol{N}_{\mathbf{prev}}^{\boldsymbol{*}}$ | $N_{prev,Min,t}<N_{\mathrm{prev}}^{*}<N_{prev,Max,t}$   \| $\boldsymbol{t}$ \| 1 \| 2 \| 3 \| 4 \| 5 \| \| --- \| --- \| --- \| --- \| --- \| --- \| \| $\boldsymbol{N}_{\mathbf{prev,Min,t}}$ \| 100 \| 110 \| 120 \| 120 \| 120 \| \| $\boldsymbol{N}_{\mathbf{prev,Max,t}}$ \| 500 \| 400 \| 300 \| 300 \| 300 \| | Ensure that transmission rates into couples and between partners generate a similar number of prevalent couples given a couple cohort of size 4,875. |
| $\boldsymbol{G}^{\boldsymbol{*}}$ | $G^{*}<G_{t}$   \| $\boldsymbol{t}$ \| 1 \| 2 \| 3 \| 4 \| 5 \| \| --- \| --- \| --- \| --- \| --- \| --- \| \| $\boldsymbol{G}_{\boldsymbol{t}}$ \| 15 \| 15 \| 10 \| 10 \| 10^1^ \| | Ensure that the proportion of couples seroconverting in each observation interval in simulations is similar to the real data. |
| ${\hat{\boldsymbol{\lambda}}}_{\mathbf{prevalent}}^{\boldsymbol{*}}$ | $m_{t}\hat{\lambda}_{\mathrm{prevalent}}$ < $\hat{\lambda}_{\mathrm{prevalent}}^{*}$ < $m_{t}^{-1}\hat{\lambda}_{\mathrm{prevalent}}$   \| $\boldsymbol{t}$ \| 1 \| 2 \| 3 \| 4 \| 5 \| \| --- \| --- \| --- \| --- \| --- \| --- \| \| $\boldsymbol{m}_{\boldsymbol{t}}$ \| 0.01 \| 0.7 \| 0.8 \| 0.85 \| 0.8^1^ \| | Ensure that the average transmission rate amongst all prevalent couples is similar to that observed in the Rakai data. |
| RH^*^_acute,univariate_ | $r_{t}\mathrm{RH}_{acute, univariate}<{\mathrm{RH}^{*}}_{acute, univariate}<r_{t}^{-1}\mathrm{RH}_{acute, univariate}$   \| $\boldsymbol{t}$ \| 1 \| 2 \| 3 \| 4 \| 5 \| \| --- \| --- \| --- \| --- \| --- \| --- \| \| $\boldsymbol{r}_{\boldsymbol{t}}$ \| 0.001 \| 0.7 \| 0.8 \| 0.83 \| 0.8^1^ \| | Ensure that the unadjusted hazard ratio between transmission within first interval incident couples and prevalent couples is similar to that observed in the Rakai data. |
| $\boldsymbol{h}^{\boldsymbol{*}}$ | $h^{*}\boldsymbol{>}11.0/7.25$ | Ensure that the simulated parameter set has enough heterogeneity to generate a ratio between unadjusted and fully (omniscient) adjusted hazard ratios that is greater than the ratio between the unadjusted and partly (non-omniscient) adjusted hazard ratios from the Wawer et al. analysis. |

^1^It was found that the criteria on ${\hat{\boldsymbol{\lambda}}}_{\mathbf{prevalent}}^{\boldsymbol{*}}$ and RH^*^_acute,univariate_ from batch 4 were overly strict and resulted in poorer fits to the Rakai data. For batch 5, we loosened these criteria and instead used a more strict criteria for *G^*^*, in which the *G* statistics for the first interval of couples’ observations were giving greater weight than other observations. These criteria correctly filtered simulations that closely

matched the observed data (S1 Fig.).
