# Supplementary material for: Reassessment of HIV-1 Acute Phase Infectivity: Accounting for Heterogeneity and Study Design with Simulated Cohorts
Source: PLoS Med. 2015 Mar 17;12(3):e1001801. doi: 10.1371/journal.pmed.1001801 (PMC4363602; doi:10.1371/journal.pmed.1001801)
Supplement: S3 Table — We generated couples cohort simulations over the entire range of parameters specified in this table. The chronic phase was defined as the period of time after the acute phase and before the late phase, and varied in duration depending on an individual’s survival time (i.e., fast progressors had shorter chronic phases). (DOCX) [file pmed.1001801.s013.docx]

S3 Table. Parameter ranges simulated. We generated couples cohort simulations over the entire range of parameters specified in this table. The chronic phase was defined as the period of time after the acute phase and before the late phase and varied in duration depending on an individual’s survival time (i.e. fast progressors had shorter chronic phases).

| Parameter | Range | Parameter | Range | References |
| --- | --- | --- | --- | --- |
| $\mathbf{RH}_{\mathbf{acute}}$ | 1 – 50 | d_acute_ | 0.5 – 6 months | [1,8,10–12] |
| $\mathbf{RH}_{\mathbf{chronic}}$ | 1 | d_chronic_ | — | — |
| $\mathbf{RH}_{\mathbf{late}}$ | 1 – 10 | d_late_ | 2 – 10 months | [1,8] |
| $\mathbf{RH}_{\mathbf{AIDS}}$ | 0 | d_AIDS_ | 10 months | [1,8] |
