# Supplementary material for: Reassessment of HIV-1 Acute Phase Infectivity: Accounting for Heterogeneity and Study Design with Simulated Cohorts
Source: PLoS Med. 2015 Mar 17;12(3):e1001801. doi: 10.1371/journal.pmed.1001801 (PMC4363602; doi:10.1371/journal.pmed.1001801)
Supplement: S4 Table — Rows in gray indicate observation intervals that were excluded from the Wawer et al. Poisson regression analysis because they were not believed to be reflective of the phase of interest (i.e., acute and late for incident and late couples, respectively). Data from all intervals were, in contrast, used by Hollingsworth et al.’s fit of a variable hazard survival model [18]. (DOCX) [file pmed.1001801.s014.docx]

S4 Table. Data from Table 1 in Wawer et al. (2005). Rows in gray indicate observation intervals that were excluded from the Wawer et al. Poisson regression analysis because they were not believed to be reflective of the phase of interest (i.e. acute or late for incident and late couples, respectively). Data from all intervals were, in contrast, used by Hollingsworth et al. (2008)’s fit of a dynamic transmission model.

| couple | interval | # infected | # not infected | total |
| --- | --- | --- | --- | --- |
| incident | 1 | 10 | 13 | 23 |
|  | 2 | 2 | 11 | 13 |
|  | 3 | 0 | 7 | 7 |
|  | 4 | 1 | 6 | 7 |
| prevalent | 1 | 14 | 147 | 161 |
|  | 2 | 9 | 120 | 129 |
|  | 3 | 10 | 82 | 92 |
|  | 4 | 3 | 42 | 45 |
| late | 4 | 2 | 20 | 22 |
|  | 3 | 9 | 26 | 35 |
|  | 2 | 8 | 23 | 31 |
|  | 1 | 0 | 13 | 13 |
