# Supplementary material for: Reassessment of HIV-1 Acute Phase Infectivity: Accounting for Heterogeneity and Study Design with Simulated Cohorts
Source: PLoS Med. 2015 Mar 17;12(3):e1001801. doi: 10.1371/journal.pmed.1001801 (PMC4363602; doi:10.1371/journal.pmed.1001801)
Supplement: S5 Table — (DOCX) [file pmed.1001801.s015.docx]

S5 Table. Assumptions made by previous analyses of the Rakai retrospective cohort that are relaxed in our re-analysis.

| Study | Assumption | Bias in EHM_acute_ | Correction |  |
| --- | --- | --- | --- | --- |
| Wawer et al. 2005 | All infections and deaths occur exactly at the midpoint of the cohort interval in which they were observed. | Slight downward | We relax this assumption (as does Hollingsworth et al.) by including a latent (unobserved) variable for infection time. |  |
| Wawer et al. 2005  Hollingsworth et al. 2008 | Incident, prevalent and late couples are *different types* of couples and real couples do *not* switch between these categories. | Slight downward | We relax this assumption by modeling in such a way that each of these categories simply represents that the cohort study only *observed* each couple in one of their disease phase categories. |  |
| Wawer et al. 2005  Hollingsworth et al. 2008 | Couples were sampled in an unbiased manner. | Substantial upward | In reality, couples providing strong evidence for lower acute phase infectivity were more likely to be excluded from the Rakai cohort based on exclusion criteria of couples lost to follow-up. We relaxed this assumption by explicitly including the study inclusion criteria in our model. |  |
| Wawer et al. 2005  Hollingsworth et al. 2008 | Transmission rates into couples and between serodiscordant partners are the same (i.e. homogenous) for all couples. | Substantial upward | We relaxed this assumption by allowing each individual to have a risk deviate that affects their risk of acquiring HIV; risk deviates were sampled from lognormal distributions with standard deviations estimated by fitting our couples transmission model to the data. |  |
