# Supplementary material for: Reassessment of HIV-1 Acute Phase Infectivity: Accounting for Heterogeneity and Study Design with Simulated Cohorts
Source: PLoS Med. 2015 Mar 17;12(3):e1001801. doi: 10.1371/journal.pmed.1001801 (PMC4363602; doi:10.1371/journal.pmed.1001801)
Supplement: S6 Table — Fig. 7 in the main text plots AFacute versus EHMacute for these studies; superscripted numbers and letters refer to the legend in Fig. 7. We do not provide confidence intervals on estimates because most studies that estimated precision used sensitivity analyses with qualitatively different justifications. (DOCX) [file pmed.1001801.s016.docx]

S6 Table. Summary of d_acute_ (duration of the acute phase), RH_acute_ (relative infectivity or hazard of acutely versus chronically infected individuals), and resulting EHM_acute_ = (RH_acute_-1)d_acute_ (excess hazard months due to the acute phase) values used in studies aiming to estimate AF_acute_ (the percentage of all transmission occurring during the acute phase). Fig. 7 in the main text plots AF_acute_ versus EHM_acute_ for these studies; superscripted numbers and letters refer to the legend in Fig. 7. We do not provide confidence intervals on estimates because most studies estimated precision used sensitivity analyses with qualitatively different justifications.

| manuscript | group | model description | d_acute_ | RH_acute_ | EHM_acute_ | AF_acute_ | parameter values from |
| --- | --- | --- | --- | --- | --- | --- | --- |
| Jacquez et al. 1994 [24] | USA  MSM^a^ | homogenous population, dynamic model | 2.0 | 80 | 158 | 25^1a^ | fits to rise in epidemic curves from San Francisco City Clinic Cohort, San Francisco Men’s Health Study, & Chicago Multicenter AIDS Cohort Study. |
|  |  |  |  | 100 | 198 | 230^1b^ |  |
|  |  |  |  | 120 | 238 | 33^1c^ |  |
|  |  |  |  | 140 | 278 | 37^1d^ |  |
|  |  | heterogenous population (three groups with different contact rates), dynamic model |  | 100 | 198 | 18^1e^ |  |
|  |  |  |  | 150 | 298 | 25^1f^ |  |
|  |  |  |  | 200 | 398 | 30^1g^ |  |
|  |  |  |  | 250 | 498 | 34^1h^ |  |
| Pinkerton & Abramson 1996 [25] | USA  MSM | 1 sex act per partner, dynamic model | 2.0 | 50 | 98 | 55^2b^ | Jacquez et al. 1994 |
|  |  |  |  | 300 | 598 | 87^2d^ |  |
|  |  | 100 sex acts per partner, dynamic model |  | 50 | 98 | 20^2a^ |  |
|  |  |  |  | 300 | 598 | 20^2c^ |  |
| Koopman et al. 1997 [26] | USA  MSM | homogenous contact, dynamic model | 1.5 | 200 | 299 | 36^3a^ | fits to rise in epidemic curves from San Francisco HBV Study. |
|  |  | age-peaked contact rate, dynamic model |  |  |  | 47^3b^ |  |
| Kretzschmar & Dietz 1998 [27] | USA  MSM | pair formation dynamic model | 1.6 | 300 | 492 | 65^4^ | Jacquez et al. 1994 |
| Xiridou et al. 2004 [23] | Europe  MSM | lower bound of Latin Hyper Cube sampling for dynamic model parameters | 1.1 | 5.8 | 9.9 | 2.7^5a^ | viral load studies |
|  |  | upper bound of Latin Hyper Cube sampling from dynamic model parameters | 4.9 | 34 | 139 | 25^5b^ |  |
| Pinkerton 2007 [28] | USA  heterosexual | static model of R0, different values are a sensitivity analysis | 1.4 | 4.2 | 4.5 | 2.5^6a^ | viral load infectivity relationship from Pilcher et al. 2004 and Rapatski et al. 2005 |
|  |  |  | 1.6 | 8.1 | 12 | 5.5^6b^ |  |
|  |  |  | 1.9 | 12 | 21 | 9.0^6c^ |  |
| Hayes & White 2006 [21] | SSA^b^  heterosexual | static model of R0, assuming an acutely infected person only had one susceptible sex partner for the rest of their life | 5.0 | 7.3 | 31 | 41^7a^ | Rakai retrospective cohort (Wawer et al.) |
|  |  | static model of R0, assuming an acutely infected person switched between multiple sex partners throughout the course of their infection (acute & chronic phases) |  |  |  | 23^7b^ |  |
| Hollingsworth et al. 2008 [18] | SSA  heterosexual | static model of R0, assuming serial monogamy | 2.9 | 26 | 73 | 9.0^8a^ | Rakai retrospective cohort (Wawer et al.), reanalyzed |
|  |  | static model of R0, assuming random mixing |  |  |  | 31^8b^ |  |
| Abu-Raddad & Longini 2008 [20] | SSA  heterosexual | dynamic model, parameterized by contact data from Kisumu, Kenya | 2.5 | 13 | 31 | 17^9a^ | Rakai retrospective cohort (Wawer et al.), reanalyzed |
|  |  | dynamic model, parameterized by contact data from Yaoundé, Cameroon |  |  |  | 25^9b^ |  |
| Salomon & Hogan 2008 [22] | SSA  heterosexual | static model of R0, assuming an acutely infected person only had one susceptible sex partner for the rest of their life | 4.0 | 8.2 | 29 | 41^10a^ | Rakai retrospective cohort (Wawer et al.) |
|  |  | static model of R0, assuming an acutely infected person switched between multiple sex partners throughout the course of their infection (acute & chronic phases) |  |  |  | 23^10b^ |  |
| Prabhu et al. 2009 [29] | USA  heterosexual | static model of R0 | 1.6 | 8.1 | 12 | 11^11^ | Pinkerton 2007 |
| Powers et al. 2011 [19] | SSA  heterosexual | dynamic model, parameterized by data from Lilongwe, Malawi | 4.8 | 30 | 141 | 38^12^ | Rakai retrospective cohort (Hollingsworth et al. 2008), reanalyzed allowing Lilongwe epidemic growth to update parameters |
| Cohen et al. 2012 (Williams) [4] | SSA  heterosexual | static model of R0 | 1.0 | 3.0 | 2.0 | 2.0^13^ | Viral load-infectivity relationships. |
| Romero-Severson et al 2013 [12] | SSA  heterosexual | individual-based episodic risk behavior model | 2 | 50 | 98 | 60^14^ | Rakai retrospective cohort ( |
| Rasmussen et al. 2014 [15] | USA  MSM | phylodynamic model | 12 | 20 | 228 | 50^15^ | phylogenetic trees used to infer transmission chains and timing |
| Bellan et al. 2015 | SSA  heterosexual | simulation-based couple transmission model | - | - | 8.4 | - | Rakai retrospective cohort (Wawer et al.), reanalyzed to take into account several biases in study design and analysis |
| Bellan et al. 2015 | SSA  heterosexual | viral load trajectories and viral load-infectivity relationships | - | - | 5.6 | - | Viral load-infectivity relationship from Lingappa et al. (2010) and viral load trajectories from Robb et al. (2012) |

**^a^**MSM; men who have sex with men

^b^SSA; sub-Saharan Africa
