# Supplementary material for: Reassessment of HIV-1 Acute Phase Infectivity: Accounting for Heterogeneity and Study Design with Simulated Cohorts
Source: PLoS Med. 2015 Mar 17;12(3):e1001801. doi: 10.1371/journal.pmed.1001801 (PMC4363602; doi:10.1371/journal.pmed.1001801)
Supplement: S1 Text — (DOCX) [file pmed.1001801.s017.docx]

**Re-assessment of HIV-1 acute phase infectivity: accounting for heterogeneity and study design with simulated cohorts**

Steve E. Bellan, Jonathan Dushoff, Alison P. Galvani, Lauren Ancel Meyers

**S1 Text**

All ‘R’ scripts used to perform the analyses in this manuscript and its supplementary material are provided in the following public GitHub repository:

<https://github.com/sbellan61/AcuteRetroSim.git>

**Table of Contents**

1. **Exclusion of Incident Serodiscordant Couples Immediately Lost to Follow-up**
2. **Couples Transmission Model**
3. **Heterogeneity in Transmission**
4. **Simulating Couple Relationship Histories**
5. **Wawer et al. Poisson Regression Model**
6. **Refitting the Hollingsworth et al. Model with Bayesian MCMC**
7. **Fitting the Couples Transmission Model with ABC-SMC**
8. **Cited References**

**I. Exclusion of Incident Serodiscordant Couples Immediately Lost to Follow-up**

In Wawer et al. (2005), the methods section states "After completion of the trial, analyses identified 414 HIV- discordant couples who subsequently received at least 1 follow- up visit that permitted the retrospective assessment of HIV transmission" [1]. Thus, only couples identified as serodiscordant couples once and who then received at least one more visit were included in the analysis. In S4 Table (Table 1 in [1]), of 23 incident couples, 10 were incident concordant positive. The above quoted statement therefore only applied to couples observed to be discordant, but was a clear misstatement with respect to these 10 concordant negative couples in which both partners seroconverted in the same follow up interval, since these couples were never observed to be discordant.

Of the 23 incident couples, 13 were incident serodiscordant and the second partner was still at risk to be observed in the next study interval. Of those 13 couples, *all* 13 were still followed up in the next study interval (the “6-15” month, i.e. second, interval in their Table 2 or our S4 Table). This means that in this retrospective cohort there were 0 couples that were seen as concordant negative, then serodiscordant, and then never observed again (at least as a couple). If such couples had been included, then the denominator in the second interval would have been less than 13 (the number of incident serodiscordant couples still at risk after the first interval).

We can estimate about how many couples were missing in a variety of ways, each of which provide similar estimates. We use the third route, which we believe to provide the most detailed data on loss to follow-up in Rakai.

1) Because 2/13 couples seroconverted in the second interval, 11 couples remained at risk for the third interval. Of those 11 couples, only 7 were observed again. This equates to a 4/11, or 36%, of couples lost after their second time being observed serodiscordant. Loss rates after their first time being observed serodiscordant are likely to have been greater, since attrition is usually greatest early in studies and because we would expect couples to dissolve or stop being involved in the study at the greatest rate the first time they realize they are serodiscordant (or at least the first time the infected partner knows their serostatus).

2) Porter et al. (2004) estimated serodiscordant couples dissolution rates (which are very high) from the same RCCS cohort data from the same time period, though she focuses on dissolution and death and doesn't look at loss-to-follow-up specifically [2].

3) Finally, we chose base our models’ parameterization of censorship on a new analysis of the Rakai Community Cohort Study. Grabowski et al. (2014) identified 135 incident couples from 1997-2011 (overlapping partially with the Wawer et al. analysis of the same cohort from 1994-1999), with couple breakdown by their serostatus and follow-up status in the Figure (right) [3]. Gray arrows show processes that caused couples to be excluded by Wawer et al.’s criteria; 59 (44%) of the couples would have been excluded by these criteria in this later Rakai cohort.

Of incident serodiscordant couples that could have been followed up (i.e. couples whose incident visit did not occur at the final visit of the study) 47% were lost to follow-up before the next visit. We use this as the probability of an incident serodiscordant couple being lost to follow-up in our simulation model, noting that additional couples will be lost to follow-up simply because their incident visit coincides with the final visit of the study.

S5 Fig. shows that in our simulations fit to the Rakai data with ABC-SMC, a median 43% (95% CI: 27-60%) of incident couples would have been excluded by the Wawer et al. criteria. This is comparable to the 44% excluded in [3], and the variation in our estimates of the proportion excluded reflects both stochastic variation in the loss to follow-up process, and also the proportion of incident serodiscordant couples whose incident serodiscordant visit was the last visit in the study period, and consequently were also excluded. Uncertainty in our posterior estimates of EHM_acute_ thus also reflect uncertainty in the exact number of couples excluded (median 17; 95% CI: 8-35).

The above figure can also be used to illustrate how excluding these incident serodiscordant couples in the original sampling design would have biased analyses of Grabowski et al. (2014)’s new data. The original sampling scheme would have calculated that 29/(29+47) = 38% of incident couples were incident concordant positive; whereas, in reality, only 29/135 = 21% were incident concordant positive.

For realism we also specified loss to follow-up rates for other serostatuses, though loss to follow-up in these couples is not problematic because it does not interact with the Wawer et al. sampling criteria in a way that causes sampling bias. Specifically, using data from Grabowski et al. (2014), we assumed that concordant negative couples and prevalent serodiscordant couples were lost to follow-up with probability 25% and 35%, respectively, during each ten-month cohort interval.

**II. Couples Transmission Model**

We fit our previously published couples transmission model to the 2004-2005 and 2011 Demographic and Health Surveys (DHS) data in Uganda [5], and for this manuscript reformulated it as a stochastic simulation of couple formation and transmission (at monthly time steps). The conceptual framework of the simulation model is similar to our previously published model, but differs in that—instead of modeling each couple’s probability of being in each serostatus type during every month of their sexually active lifetime (i.e. a Markov chain state probability model)—transmission is modeled as a discrete process with HIV infection, AIDS mortality, and couples aging out of the cohort occurring as discrete events in time.

For each simulation, we generated couple relationship histories (i.e., sexual debuts, couple formation dates, and ages) using the correlated multivariate distributions of these variables estimated from the DHS (Section IV of this text; S8 Fig.). HIV transmission was modeled independently for each simulated couple as follows. In each month of these relationship histories, sexually active susceptible partners could become infected with a probability determined by the corresponding hazard rates. We specified three possible routes of infection:

1. prior to formation of the current partnership (pre-couple transmission),
2. during their partnership but from sources other than the current partner (extra-couple transmission),
3. during the partnership by their partner (within-couple transmission).

Male and female partners experience risk from pre-couple transmission starting in the month of their sexual debuts up until the month before they form into a couple. Each partners’ probability of infection in each month is calculated using the rate to probability transformation $p_{M,b,i,t}=1-exp(-\lambda_{M,b,i,t}\Delta t)$ where $\Delta t$ is one month and $\lambda_{M,b,i,t}$ is the pre-couple (b as in ‘b’efore) transmission hazard experienced by the male partner in the *i*-th couple for transmission at month *t* (for females we index ‘f’ instead of ‘m’). Similarly, male partners are infected via extra-couple transmission from external partners during their stable partnership with probability $p_{M,e,i,t}=1-exp(-\lambda_{M,e,i,t}\Delta t)$. Male partners are infected by their stable female partners during their relationship with probability $p_{M,p,i,t}=1-exp(-\lambda_{M,p,i,t}\Delta t)$.

The hazards used to calculate these monthly probabilities of infection change over time with changing population prevalence (pre-couple and extra-couple routes) and depending on partner infection status and disease stage (within-couple route). Specifically, pre-couple and extra-couple hazards are functions of the time-varying prevalence in the opposite gender’s population,

$$\lambda_{M,b,i,t}=\beta_{M,b,i}{\times P}_{F}\left( t \right)$$

$$\lambda_{F,b,i,t}=\beta_{F,b,i}{\times P}_{M}\left( t \right)$$

$$\lambda_{M,e,i,t}=\beta_{M,e,i}{\times P}_{F}\left( t \right)$$

$\lambda_{F,e,i,t}=\beta_{F,e,i}{\times P}_{M}\left( t \right)$,

where $P_{M}\left( t \right)$ and $P_{F}\left( t \right)$ indicate the male and female population infectious HIV prevalence (proportion infected and *not* on ART) in Uganda at time *t*, respectively (taken from UNAIDS [6]), and each $\beta$ is a transmission coefficient reflective of the probability of transmission per coital act and sexual networking behavior. We can separate these parameters further, for example, as $\beta_{M,b,i}=\beta_{M,i}^{*} c_{M,b,i}$, where $\beta_{M,i}^{*}$ reflects the transmission rate experienced by the *i*-th male partner and $c_{M,b,i}$ reflects his sexual contact patterns prior to couple formation. In contrast, within-couple transmission is dependent on partner infection status,

$$\lambda_{M,p,i,t}=\left\{ \begin{aligned} 0 if partner uninfected \\ \beta_{M,i}^{*}\mathrm{RH}_{F,i,t} if partner infected \end{aligned} \right.$$

$$\lambda_{F,p,i,t}=\left\{ \begin{aligned} 0 if partner uninfected \\ \beta_{F,i}^{*}\mathrm{RH}_{M,i,t} if partner infected \end{aligned} \right.$$

where ${RH}_{M,i,t}$ and ${RH}_{F,i,t}$ reflect the relative hazard of an individual’s sexual partner at time *t* relative to the chronic stage. Specifically,

$$\mathrm{RH}_{M,i,t}=\left\{ \begin{aligned} \begin{matrix} \mathrm{RH}_{\mathrm{acute}} & \text{if the }\text{i-}\text{th}\text{ male partner is in the acute stage at time }\text{t} \end{matrix} \\ \begin{matrix} 1 & \text{if the }\text{i-}\text{th}\text{ male partner is in the chronic stage at time }\text{t} \end{matrix} \\ \begin{matrix} \begin{matrix} \mathrm{RH}_{\mathrm{late}} & \text{if the }\text{i-}\text{th}\text{ male partner is in the late stage at time }\text{t} \end{matrix} \\ \begin{matrix} 0 & \text{if the }\text{i-}\text{th}\text{ male partner is in the AIDS stage at time }\text{t} \end{matrix} \end{matrix} \end{aligned} \right.$$

and similarly for $\mathrm{RH}_{F,i,t}$ reflecting the female partner’s disease stage.

Once infected, individuals are assigned date of death due to AIDS mortality by sampling a random variable from the Weibull distribution corresponding to the age-at-seroconversion dependent survival functions previously described [5,7]. Then, we define an individual’s disease progression by an acute phase lasting d_acute_ months after infection. The acute phase is then followed by the chronic phase, which continues until (d_late_ + d_AIDS_) months before death upon which the late phase begins. The late phase then lasts until d_AIDS_ months prior to death. After the late phase, the individual is in the AIDS phase for the final d_AIDS_ months of their life, during which they are assumed to be too sick to have sex with and infect their partner.

In simulating the times of death from the Weibull model, a sampled survival time occasionally (but very infrequently) was less than d_acute_ + d_late_ + d_AIDS_. When, this occurred, we used the following priority rules:

- The first d_acute_ months after infection are always the acute phase.
- The last d_AIDS_ months before death are always the AIDS phase unless some of these months qualified to be the acute phase.
- The d_late_ months prior to the AIDS phase are the late phase unless some of these months qualified to be the acute phase.

In our ABC-SMC analysis, we fit all transmission rates and acute phase infectivity and duration. For our simulation analysis, we varied phase relative infectivity and duration parameters across the ranges specified in S3 Table; we further assumed the population mean within-couple transmission rates to be those values estimated from Rakai [8] ($\bar{\beta}_{M}^{*}=\bar{\beta}_{F}^{*}=$0.007 per month; similar to our DHS estimates of 0.0077), and pre-couple and extra-couple transmission rates that we previously estimated from DHS data ($\bar{\beta}_{M,b}=0.0093$, $\bar{\beta}_{F,b}=0.022$, $\bar{\beta}_{M,e}=0.0038$, $\bar{\beta}_{F,e}=0.0047$ [5]; note that these are transmission coefficients—i.e. prevalence-standardized hazards—and equate to the hazard after being multiplied by the prevalence; see above). Individual transmission coefficients and hazards varied around population median values as described in Section III below (Heterogeneity in Transmission).

Our previous manuscript explains in detail how we estimated route-specific transmission coefficients above from the DHS [5]. Briefly, we estimated the posterior distributions of the six transmission coefficients ($\bar{\beta}_{M,b}$, $\bar{\beta}_{F,b}$, $\bar{\beta}_{M,e}$, $\bar{\beta}_{F,e}$, $\bar{\beta}_{M}^{*}$, $\bar{\beta}_{F}^{*}$,) that best fit the couples’ data. Model fitting leveraged information from relationship histories to estimate these transmission coefficients. For instance, infected partners in serodiscordant couples that were formed more than 15 years prior to testing serodiscordant were almost certainly infected extra-couply, because, if infected pre-couply, they would not have been alive at the time of the DHS survey (ART coverage was low during the period under consideration). Infected partners in serodiscordant couples whose sexual debut occurred at (or shortly before) couple formation with their current partner were also almost certainly infected extra-couply because they could not have been infected prior to couple formation since they were not then sexually active. Prevalence of infection relative to the period of sexually activity prior to couple formation also informs the rate of pre-couple transmission. Similarly, the changing ratio of serodiscordance to concordance positivity with increasing couple duration informs estimates of within-couple transmission rates. While we used these DHS estimated values for our simulation analysis, in our ABC-SMC analysis, we held late and AIDS phase characteristics constant while estimating all nine other transmission, acute phase, and heterogeneity parameters directly from the Rakai data (Section VII).

**III. Heterogeneity in Transmission**

Heterogeneity was included in the model by allowing each individual to experience their own transmission rate. In a homogenous model, each individual experiences the same transmission hazard,

$$\beta_{M,i}^{*}=\bar{\beta}_{M}^{*}$$

$\beta_{F,i}^{*}=\bar{\beta}_{F}^{*}$.

In a heterogeneous model, we allow these transmission hazards to vary log-normally around the population mean by multiplying the population mean by a log-normally distributed risk deviate with an expected value of 1. To avoid dealing with the less familiar parameterization of the lognormal distribution itself, we chose to parameterize this distribution on the scale of the normal distribution and to then transform to a log-normally distribution. Male and female partners have risk deviates $Z_{M,i}$ and $Z_{F,i}$ distributed as

$$\log\left( Z_{M,i} \right)\mathcal{\sim N(-}\frac{\sigma^{2}}{2},\sigma)$$

$$\log\left( Z_{F,i} \right)\mathcal{\sim N(-}\frac{\sigma^{2}}{2},\sigma)$$

which yields $Z_{M,i}$ and $Z_{F,i}$, each with an expected value (mean) of 1. We can then describe the transmission rate experienced by the *i*-th male and female partners as

$$\beta_{M,i}^{*}={Z_{M,i}\bar{\beta}}_{M}^{*}$$

$\beta_{F,i}^{*}=Z_{F,i}\bar{\beta}_{F}^{*}$,

We are primarily interested in heterogeneity in the transmission rate between stable partners because this can affect retrospective cohort data like that acquired in Rakai. We assume that heterogeneity in individuals’ pre-couple and extra-couple sexual contact rates do not interact with within-couple transmission. In other words, we assume that the partnering behavior of an individual before couple formation or with extra-couple partners is not related to the rate at which they infect their partner *given* that they have been infected. We therefore ignore this heterogeneity in the contact coefficients in this analysis and assume that each contact rate equals the population average (e.g., $c_{M,b,i}=\bar{c}_{M,b}$ and equivalently $\beta_{M,b,i}=\bar{\beta}_{M,b}$). This yields the following hazards for each *i*-th individual and route,

$$\lambda_{M,p,i,t}=\left\{ \begin{aligned} 0 if partner uninfected \\ {Z_{M,i}\bar{\beta}}_{M}^{*}\mathrm{RH}_{F,i,t} if partner infected \end{aligned} \right.$$

$$\lambda_{F,p,i,t}=\left\{ \begin{aligned} 0 if partner uninfected \\ Z_{F,i}\bar{\beta}_{F}^{*}\mathrm{RH}_{M,i,t} if partner infected \end{aligned} \right.$$

$$\lambda_{M,b,i,t}=Z_{M,i}\bar{\beta}_{M,b}P_{F}\left( t \right)$$

$$\lambda_{F,b,i,t}=Z_{F,i}\bar{\beta}_{F,b}P_{M}\left( t \right)$$

$$\lambda_{M,e,i,t}=Z_{M,i}\bar{\beta}_{M,e}P_{F}\left( t \right)$$

$$\lambda_{F,e,i,t}= Z_{F,i}\bar{\beta}_{F,e}P_{M}\left( t \right)$$

Because each individual hazard is the product of a population hazard and a risk deviate with expected value of 1, we can also write that the hazard of individual from the *r*-th route of transmission as

$$\log\left( \lambda_{M,r,i,t} \right)\mathcal{\sim N}\left( \log\left( \dot{\lambda}_{M,r,t} \right)-\frac{{\sigma_{\mathrm{hazard}}}^{2}}{2},\sigma_{\mathrm{hazard}} \right)$$

$$\log\left( \lambda_{F,r,i,t} \right)\mathcal{\sim N}\left( \log\left( \dot{\lambda}_{F,r,t} \right)-\frac{{\sigma_{\mathrm{hazard}}}^{2}}{2},\sigma_{\mathrm{hazard}} \right),$$

which yields $\lambda_{M,r,i}$ and $\lambda_{F,r,i}$ with expected values of $\dot{\lambda}_{M,r}$ and $\dot{\lambda}_{F,r}$. Elsewhere, we refer to the mean chronic phase transmission rate as $\dot{\lambda}_{\mathrm{hazard}}$ and, for brevity, do not differentiate between sex-specific rates. While these values vary between route and over time (with population prevalence or stable partner infection status and disease stage), we chose to describe our implementation of heterogeneity defined above more simply in the main text as

$$\lambda{}_{i}\sim\mathrm{logNormal}\left( \bar{\lambda}_{\mathrm{hazard}},\sigma_{\mathrm{hazard}} \right),$$

to highlight that all transmission rates vary log-normally around a population median ($\bar{\lambda}_{\mathrm{hazard}}$; which is the exponent of the log-scale population mean).

Variation between individuals in this risk deviate corresponds to any persistent difference in the rate at which they get infected when exposed to an infectious person. While these clearly include differences in susceptibility (due to e.g. differences in genetics, sexual behavior, circumcision status, immune system competence), these can also include differences in a stable partner’s infectiousness in our model since any increase in infectiousness would affect the same hazard term. Thus, rather than include separate risk deviates corresponding to uninfected partner susceptibility and infected partner infectiousness, we use these $Z$ deviates to reflect the differences between couples. $Z_{M,i}$ then reflects the relative rate at which male partners are infected by their female partner (if she is infected) in the *i*-th couple and conveys information about both the male partner’s susceptibility relative to the population mean and the female partner’s infectiousness relative to the population mean. Consequently, $\sigma_{\mathrm{hazard}}$ aggregates inter-couple variation in couple level variables such as frequency of unprotected sex or intercourse type (vaginal/anal); in uninfected partner variables such as genetic resistance, partial immunity due to previous exposure, co-infections, or circumcision; or in infectious partner variables such as co-infections, viral genetics, or viral load.

We fit $\sigma_{\mathrm{hazard}}$, directly to the Rakai data to determine the appropriate range over which to simulate in our simulation analysis (see Section VII). We estimated $\sigma_{\mathrm{hazard}}$ = 2.0 (1.2 – 2.8). Our results were consistent with the substantial amount of heterogeneity estimated in the Hughes et al. (2011) analysis of the Partners in Prevention serodiscordant couple cohort. They fit couple-level random effects in the per-coital-act infectivity of chronically infected individuals phase and estimated $\sigma_{\mathrm{hazard}}$ = 3.25 (personal communication of unpublished values from [32]). Measured variability in viral load also supports the plausibility of such high values. In a sample of 39 individuals repeatedly sampled for 100 days post-infection, individual acute phase peak and chronic phase set point viral loads spanned a range of 4 and 4.5 log10 units, respectively, with peak and set point viral loads highly correlated [30]. Based on the viral load infectivity relationship of a 2.5 increase per log10 unit [3,43], this 4.5 log10 viral load variation in the chronic phase alone may drive about a 50-fold difference in infectivity between the least and most infectious individuals in this small sample.

Based on our fitted results above, in our simulation analyses we simulated data with hazards drawn from log-normal distributions with $\sigma_{\mathrm{hazard}}=$1, 2, or 3 correspond to the 97.5 percentile individuals experiencing infection hazards 7, 50, or 360 times greater than $\bar{\lambda}_{\mathrm{hazard}}$, respectively. In our simulation analysis, we also simulated ‘observed covariates’ for each individual that partially correlated with their risk deviate and included these in multivariate regressions to assess how estimated EHM_acute_ is affected by controlling for some, but not all, of individual heterogeneity through covariates as those included by Wawer et al. (e.g. self-reported genital ulcer, coital rate). For male partner *i*, their risk deviate is given by *Z*_M,_*_i_* as described above. We denote this individual’s measured covariate as *W*_M,_*_i_* and sample this variable conditional on their underlying true risk, using the formula for sampling a normal random variable conditional on another normal random variable with known correlation between the variables $C$,

$\log\left( W_{M,i} \right)\mathcal{\sim N}\left( C\log\left( Z_{M,i} \right),\sqrt{\left( \sigma_{\mathrm{hazard}} \right)^{2}-C^{2}\left( \sigma_{\mathrm{hazard}} \right)^{2}} \right)$.

Thus, if $C=0.7$ then $\log\left( W_{M,i} \right)$ is sampled conditional on $\log\left( Z_{M,i} \right)$ such that they have a correlation coefficient of 0.7. By controlling for $\log\left( W_{M,i} \right)$ in a multivariate Poisson regression model where the underlying hazards are scaled by $Z_{M,i}$, we can then control for 0.49 of the variance between individuals.

**IV. Simulating Couple Relationship Histories**

We developed methods to simulate relationship histories so that we could simulate sufficiently large populations to disentangle systematic biases in EHM_acute_ estimators from noise due to small sample sizes. While relationship histories (ages and timing of each partner’s sexual debut, time of couple formation) are directly available from the DHS, we wanted to account for the survival bias inherent in cross-sectional data sets such as DHS (i.e., the only couples that can be sampled by such surveys are those for which both members are alive at the time of sampling [5]). Without accounting for this bias, we would be initializing simulations at the beginning of the HIV epidemic in a biased population of couples that were more likely to live up until a recent DHS survey.

Thus, we created two methods to simulate relationship histories representative of those in the Ugandan DHS and adjusted for survivor bias. The first method extracts a multivariate signature of the five key relationship variables for each couple (ages at sexual debuts for men and women, time between sexual debut and couple formation for each gender, and date of couple formation) using a Gaussian copula model and then uses these same models to randomly generate new couples with similar relationship patterns. The second method is based on replicating DHS-observed couples’ relationship histories based on their inverse probability of surviving to DHS sampling.

The choice of method did not affect our results and so we chose to use the Gaussian copula approach because it more realistically reflects the continuous nature of relationship history variables.

*Gaussian Copula Approach*

We then needed to generate populations of couples with realistic relationship histories (ages and dates of sexual debuts and couple formation) in which to simulate the transmission dynamics above. We developed methods to synthesize populations of couples by extracting a multivariate signature of five key relationship variables for each couple (ages at sexual debuts for men and women, time between sexual debut and couple formation for each gender, and date of couple formation) for each country using Gaussian copula models and then uses these same models to randomly generate new couples with similar relationship patterns. The distribution of these five variables exhibits nontrivial correlations within each country-group data set, and their multivariate distributions differ greatly between countries. Specifically, to generate couples with relationship histories representative of observed couples we:

1. Fit a continuous kernel density function to the empirical distribution of each of the five relationship variables using either the ‘density’ or ‘logspline’ functions in R weighting each couple by their inverse probability of surviving to be sampled in the Ugandan DHS [5] and choosing the smoothing bandwidth empirically based on visual inspection of the fit to histograms.
2. Discretize the fitted density function into monthly intervals to create a probability distribution function (PDF) for each of the five random variables. The resulting PDF for age at male sexual, for example, could then generate random ages at male sexual debut representing the real data. However, these five PDFs are still independent and in the next steps multivariate correlation structures are fitted.
3. Transform these PDFs into cumulative density functions (CDFs).
4. Use these CDFs to transform the observations of these five variables for each couple to their quantile on [0,1].
5. Use the normal quantile function ‘qnorm’ to transform each of these quantiles to their corresponding value on (-∞, ∞).
6. Use ‘cov.wt’ to get the covariance-variance matrix for these five variables where each couple is weighted by their inverse probability of surviving to be observed in the DHS sample.

Then, to generate 100,000 couples with the above covariance-variance structure:

1. Generate 100,000 correlated multivariate normal vector of dimension five with the above covariance-variance structure.
2. Transform these variables to their quantiles on [0, 1] using the normal CDF (‘pnorm’).
3. Use the inverse CDFs created in step (3) above to transform these correlated quantile vectors back to discrete monthly vectors with the observed correlation structure.

S8 Fig. compares pairwise plots for the five relationship variables both for the observed data from the Ugandan DHS and our fitted copula model.

*Non-parametric Sampling-Based Approach*

In the non-parametric approach, simulated couple populations were constructed by calculating the probability that couples observed in the DHS survived to their interview time (as calculated using our fitted couple transmission model [5]). The inverse of these probabilities were then use to weight the occurrence of couples in the simulated population. We simulated large couple populations relative the size of the DHS sample populations by replicating each observed couple $X_{i}$ times where $X_{i} \sim Poisson(\frac{S}{Z_{i}})$, $Z_{i}$ is the probability that both members of the *i*-th couple survived until their DHS interview, and S is a scalar that increases the number of couples such that a much larger population can be created. We choose S such that the resulting population is well over 100,000 and then randomly sample 100,000 pseudo-couples from that population to yield our final representative population.

**V. Wawer et al. Poisson Regression Model**

For each simulated retrospective cohort, we replicated the approach of Wawer et al. (2005) and estimated RH_acute_ using a Poisson regression of second partner seroconversion against index partner disease phase, with second partner person-time at risk included as an offset term. Person-time at risk was calculated by assuming that infections or deaths occurring in a 10-month interval occurred at the 5-month midpoint of interval; when both partners were infected in the same interval, the second individual was assumed to have been infected at 7.5 months [1].

In prevalent couples, it was assumed that the index partner was chronically infected throughout the entire period of observation. Consequently, all prevalent couples’ observation intervals were included in the regression as representative of chronic phase exposure. In contrast, because the acute phase was assumed to only last a few months, Wawer et al. assumed the index partner’s acute phase was entirely contained during which they were infected, and that during subsequent intervals, the index partner was chronically infected. Thus, for incident couples, only the interval of index partner infection was used as representative of acute phase exposure. Given the person-time assumptions above, this interval represents the second partner’s exposure to the index partner during 0 – 5 months after the latter’s infection, implicitly assuming that the acute phase lasts 5 months.

Similarly, Wawer et al. assumed that during the interval preceding a partner’s death that they were too sick to transmit (i.e. AIDS phase). Because their unadjusted hazard calculations were greater for the two penultimate 10-month intervals, they further assumed that only these intervals constituted the late phase. Thus, only the second and third to last intervals (6 – 25 months prior to partner death by the midpoint assumptions) before partner death from late couples were included in the regression [1].

**VI. Refitting the Hollingsworth et al. Variable Hazard Survival Model with Bayesian MCMC**

Hollingsworth et al. (2008) fit a variable hazard survival model to the retrospective cohort data [8] published by Wawer et al. (2005) to estimate the hazards (i.e. infectivity per unit time) of the acute, chronic, and late phases as well as the duration of the acute, late and AIDS phases (where the latter is defined as the period prior to death when infected individuals are too sick to have sex with and expose their partners).

We write out the likelihoods for each couple class below. Couples are observed at intervals of length *T* = 10 months. For incident serodiscordant couples, we define the hazard of transmission as a function of time since the index partner’s time of infection. This timing is unknown, so Hollingsworth et al. (2008) assume that it occurs at a hidden unknown event time with equal probability of having occurred at any time in the interval of occurrence. The hazard can then be written as a function of time since index partner infection, *t*,

$$\beta_{\mathrm{inc}}\left( t \right)=\left\{ \begin{matrix} \beta_{\mathrm{acute}}\text{ if }\text{t}\text{ ≤ }d_{\mathrm{acute}} \\ \beta_{\mathrm{chronic}}\text{ if }\text{t}\text{ > }d_{\mathrm{acute}} \end{matrix} \right..$$

We can then write the probability than the index-infected partner infected their partner within the same interval as their own infection as

$$p_{\mathrm{inc},1}=\int_{0}^{T} \left( 1-\exp\left( -\int_{0}^{T-t_{\mathrm{index}}} \beta_{\mathrm{inc}}\left( t \right)dt \right) \right)dt_{index},$$

where *t*_index_ denotes the unknown time of the index partner’s infection since the couples’ last observation as concordant negative (i.e. 0 < *t*_index_ < *T*). The probability that they infect their partner in the second or later interval (i.e., the *j*-th interval) after they were observed concordant negative is given by

$$p_{\mathrm{inc},j}=\int_{0}^{T} \left( 1-\exp\left( -\int_{\left( j-1 \right)T-t_{\mathrm{index}}}^{jT-t_{\mathrm{index}}} \beta_{\mathrm{inc}}\left( t \right)dt \right) \right)dt_{index},$$

The probability that transmission occurs between partners in a ‘prevalent’ serodiscordant couple is given by

$$p_{\mathrm{prev}}=1-\exp\left( -\beta_{\mathrm{acute}}T \right).$$

For late couples, the hazard of transmission is given as a function of time prior to the index-infected partner’s death, *t*,

$$\beta_{\mathrm{late}}\left( t \right)=\left\{ \begin{matrix} 0\text{ if }\text{t}\text{ ≤ }d_{\mathrm{AIDS}} \\ \begin{matrix} \beta_{\mathrm{late}}\text{ if }d_{\mathrm{AIDS}}\text{<}\text{t}\text{ ≤ }d_{\mathrm{AIDS}}+d_{\mathrm{late}} \\ \beta_{\mathrm{chronic}}\text{ if }\text{t}\text{ > }d_{\mathrm{AIDS}}+d_{\mathrm{late}} \end{matrix} \end{matrix} \right..$$

The probability of transmission in the same interval as death is then given by

$$p_{\mathrm{late},1}=\int_{0}^{T} \left( 1-\exp\left( -\int_{0}^{T-t_{\mathrm{death}}} \beta_{\mathrm{late}}\left( t \right)dt \right) \right)dt_{\mathrm{death}},$$

where *t*_death_ denotes the time between the index partner’s death and the following survey visit—such that there was *T* – *t* time in the interval during which they died that they could have exposed their partner. The probability of transmission in the *j*-th interval prior to the first observation in which the partner was dead is given by

$$p_{\mathrm{late},j}=\int_{0}^{T} \left( 1-\exp\left( -\int_{\left( j-1 \right)T-t_{\mathrm{death}}}^{jT-t_{\mathrm{death}}} \beta_{\mathrm{late}}\left( t \right)dt \right) \right)dt_{\mathrm{death}}.$$

S4 Table replicates the data from Table 1 in Wawer et al. (2005) where interval #’s represent the interval after the last observation as concordant negative (incident couples), the interval after the first observation as serodiscordant (prevalent couples), or the interval prior to the couples’ first observation with the index partner dead (e.g. 1 corresponds to the interval during which they died and 2 corresponds to the preceding one).

We can fit the Hollingsworth model by noting that the likelihood is the probability each couple either was or was not infected in each interval of observation. The likelihoods of any parameterization for incident, prevalent, and late couples are then given by

$$L_{\mathrm{inc}}=\left( p_{\mathrm{inc},1} \right)^{10}\left( p_{\mathrm{inc},2} \right)^{2}\left( p_{\mathrm{inc},3} \right)^{0}\left( p_{\mathrm{inc},4} \right)^{1}\left( 1-p_{\mathrm{inc},1} \right)^{13}\left( 1-p_{\mathrm{inc},2} \right)^{11}\left( 1-p_{\mathrm{inc},3} \right)^{7}\left( 1-p_{\mathrm{inc},4} \right)^{6},$$

$$L_{\mathrm{prev}}=\left( p_{\mathrm{prev}} \right)^{14+9+10+3}\left( 1-p_{\mathrm{prev}} \right)^{147+120+82+42}$$

$$L_{\mathrm{late}}=\left( p_{\mathrm{late},4} \right)^{2}\left( p_{\mathrm{late},3} \right)^{9}\left( p_{\mathrm{late},2} \right)^{8}\left( p_{\mathrm{late},1} \right)^{0}\left( 1-p_{\mathrm{late},4} \right)^{20}\left( 1-p_{\mathrm{late},3} \right)^{26}\left( 1-p_{\mathrm{late},2} \right)^{23}\left( 1-p_{\mathrm{late},1} \right)^{13},$$

and the full model likelihood is given by

$$L(\beta_{\mathrm{chronic}}, \mathrm{RH}_{\mathrm{acute}},d_{\mathrm{acute}},\mathrm{RH}_{\mathrm{late}},d_{\mathrm{late}},d_{\mathrm{AIDS}}|data)=L_{\mathrm{inc}}L_{\mathrm{prev}}L_{\mathrm{late}}.$$

We write the likelihood for the original Rakai retrospective cohort data set above to allow specific numbers to better build intuition for its meaning. However, this same likelihood can be applied to fit this model to any of our simulated retrospective cohorts.

We fitted this variable hazard survival model to estimate its parameters by sampling the posterior parameter distribution with Bayesian Markov Chain Monte Carlo (MCMC) using a Metropolis-Hastings algorithm. For each fit, we ran 12 chains in parallel. All six parameters being sampled (acute to chronic relative hazard, RH_acute_; late to chronic relative hazard, RH_late_; chronic hazard, $\beta_{\mathrm{chronic}}$; acute phase duration, d_acute_; late phase duration, d_late_; AIDS phase duration, d_AIDS_) were sampled on a log-scale and given uninformative uniform priors on that scale. Therefore, numerical integration of the posterior equated to numerical integration of the likelihood written above (ratios between prior probabilities for current and proposed parameters always equaled 1). Chains were initialized at random initial values for each parameter. We then performed an adaptive sample of length 5000 with a burn-in of 1000 iterations and no thinning. We used blocked multivariate normal sampling for all six parameters, with a best guess of a covariance-variance matrix for the multivariate normal proposal distribution for this first adaptive phase. From these resulting samples, we then calculated an empirical covariance-variance matrix and repeated an adaptive sample of the same length with new random initial values. We then recalculated the empirical covariance-variance matrix from this second adaptive phase and used it in a final sampling phase of length 10,000 iterations with a burn-in of 1,500 iterations (again starting from new random initial conditions). The acceptance ratio was close to the optimal value for multivariate block sampling using this algorithm (about 0.18). We calculated the Gelman-Rubin diagnostic to check for convergence and also visually inspected a subset of iteration trace plots. Results were then calculated as parameter medians and 95% credible intervals (both for parameters listed above and derived parameters such as EHM_acute_). In the case of Fig. 1, we calculated a 95% contour using the 95% sample volume as estimated by a kernel density approach (with the HPDregionplot function in R package ‘emdbook’).

**VII. Generating data with the Hollingsworth Model**

Because the Hollingsworth Model is a fully specified probability model, we can simulate couple cohort data with the same model. Simulating data with the model clarifies the model assumptions as well as allows us to validate our model fitting algorithm—fitting the model to data generated by itself should also recover the original parameters (for sufficiently large sample sizes) unless the fitting algorithm is biased or has a bug.

To simulated with the Hollingsworth model we specified the total number of couples and each couple “class” as N_inc_, N_prev_, N_late_, for incident, prevalent, and late couples, respectively. Then, for each incident couple we picked at time of infection within the interval of infection, such that the time of infection at a uniform probability of occurring at each time an interval:

$$t_{\mathrm{index}}\sim\mathrm{Uniform}\left( 0,T \right).$$

We then calculated the remaining time in this interval as ($T-t_{\mathrm{index}})$, and calculated the probability that the index-infected individual infected their partner as

$$p_{\mathrm{inc},1}=1-\exp\left( -\int_{0}^{T-t_{\mathrm{index}}} \beta_{\mathrm{inc}}\left( t \right)dt \right).$$

If the second partner is not infected in this same interval as the index-infected partner, then they can be infected in each of the subsequent intervals (conditional on not being infected in each previous interval) with probability,

$$p_{\mathrm{inc},j}=1-\exp\left( -\int_{\left( j-1 \right)T-t_{\mathrm{index}}}^{jT-t_{\mathrm{index}}} \beta_{\mathrm{inc}}\left( t \right)dt \right).$$

Similarly, for prevalent and late couples, the probability of infection during each interval is given by

$$p_{\mathrm{prev}}=1-\exp\left( -\beta_{\mathrm{acute}}T \right),$$

and

$$t_{\mathrm{death}}\sim\mathrm{Uniform}\left( 0,T \right)$$

$$p_{\mathrm{late},1}=1-\exp\left( -\int_{0}^{T-t_{\mathrm{death}}} \beta_{\mathrm{late}}\left( t \right)dt \right)$$

$$p_{\mathrm{late},j}=1-\exp\left( -\int_{\left( j-1 \right)T-t_{\mathrm{death}}}^{jT-t_{\mathrm{death}}} \beta_{\mathrm{late}}\left( t \right)dt \right).$$

Thus, we use Bernoulli random sampling to determine the probability that transmission occurs within a serodiscordant couple of each type. Notably, in this model there is no loss to follow-up and couples do not switch between classes—a couple is a priori considered ‘incident’, ‘prevalent’ or ‘late’—meaning that this model does not simulate misclassification of couples by disease stage as actually occurs in reality when using observed states as indicators of disease progression. We were able to fit the Hollingsworth Model to data generated by this same model using the above algorithm with high accuracy (S4 Fig.).

**VIII. Fitting the Couples Transmission Model with ABC-SMC**

*Parameters Fit with ABC-SMC*

Approximate Bayesian computation with sequential Monte Carlo is a statistical method of fitting a simulation model to data to estimate the model’s parameters [8]. We used ABC-SMC to estimate nine parameters by fitting our couple simulation model to the Rakai cohort data. These parameters included the six gender-route specific transmission rates ($\bar{\beta}_{M,b}$, $\bar{\beta}_{F,b}$, $\bar{\beta}_{M,e}$, $\bar{\beta}_{F,e}$, $\bar{\beta}_{M}^{*}$, $\bar{\beta}_{F}^{*}$), the acute to chronic phase relative hazard (RH_acute_), the acute phase duration (d_acute_), and the inter-individual heterogeneity in risk ($\sigma_{\mathrm{hazard}}$). We note here that in our ABC-SMC procedure, we choose a more stable parameterization of the six gender-route specific transmission coefficient parameters that uses geometric mean coefficient between genders,

$\bar{\beta}_{b}=\left( \bar{\beta}_{M,b}\bar{\beta}_{F,b} \right)^{\frac{1}{2}}$,

$\bar{\beta}_{e}=\left( \bar{\beta}_{M,e}\bar{\beta}_{F,e} \right)^{\frac{1}{2}}$,

$\bar{\beta}^{*}=\dot{\lambda}_{\mathrm{hazard}}=\left( \bar{\beta}_{M}^{*}\bar{\beta}_{F}^{*} \right)^{\frac{1}{2}}$,

and male to female transmission coefficient ratios,

$\rho_{b} =\frac{\bar{\beta}_{M,b}}{\bar{\beta}_{F,b}}$,

$\rho_{e} =\frac{\bar{\beta}_{M,e}}{\bar{\beta}_{F,e}}$,

$\rho^{*}=\frac{\bar{\beta}_{M}^{*}}{\bar{\beta}_{F}^{*}}$.

Fitting the transmission coefficients as geometric means and sex ratios allowed us to use informative priors on the transmission coefficient sex ratios to avoid independently sampling male and female transmission coefficients, which could lead to orders of magnitude difference in transmission to either sex. Thus, the parameter vector fitted to the data was $\theta=\{\bar{\beta}_{b},\bar{\beta}_{e},\bar{\beta}^{*}, \rho_{b},\rho_{e},\rho^{*},\mathrm{RH}_{\mathrm{acute}}, d_{\mathrm{acute}},\sigma_{\mathrm{hazard}}\}$. We used informative lognormal priors on the three transmission coefficient sex ratio parameters ($\rho_{b},\rho_{e},\rho^{*}$), based on posterior estimates for these parameters from our fitted couple transmission model to Uganda DHS data [4]. For all other parameters, we used uninformative uniform prior distributions on a log scale ($\bar{\beta}_{b},\bar{\beta}_{e},\bar{\beta}^{*}$, RH_acute_, d_acute_) or an untransformed scale ($\sigma_{\mathrm{hazard}}$), with upper and lower bounds chosen to be wide enough to avoid boundary effects on the posterior distribution using preliminary ABC-SMC analyses. Upper bounds on $\bar{\beta}^{*}$ had to be surprisingly high to allow the model to fit the data when $\sigma_{\mathrm{hazard}}$ was high. In other words, if individuals’ transmission risk is extremely heterogeneous, then transmission rates measured in prevalent couples (who are selected for low within-couple transmission rates) are extremely unrepresentative of the mean transmission rate in the general population, which is necessarily much higher. We set the following as fixed parameters: RH_late_ = 5, RH_AIDS_ = 0, and d_late_ = 10, d_AIDS_ = 10, following Hollingsworth et al.’s results. We fixed these parameters because the goal of this fitting procedure is to estimate acute phase characteristics, and because we found previous estimates of late and AIDS phase parameters [7] to be robust in our replication of previous analyses on simulated data.

*ABC-SMC Fitting Procedure*

We used the following ABC-SMC algorithm [8] to create an approximate posterior distribution

1. Set $t=1$.
   1. If $t=1$, Sample $\theta^{**}$ **(**i.e., a particle) from the prior $\pi(\theta)$ (S1 Table).
   2. Else, sample $\theta^{*}$ from the previous population {$\theta_{t-1}^{\left( i \right)}\}$ with weights $w_{t-1}$ and perturb the particle to obtain $\theta^{**}\sim K_{t}(\theta|\theta^{*})$. We use a uniform perturbation kernel with half-widths equal to the standard deviation {$\theta_{t-1}^{\left( i \right)}\}$. This yields $K_{t}\left( \theta| \theta^{*} \right)= \mathrm{unif}(\theta^{*}-\sigma_{t-1}, \theta^{*}+\sigma_{t-1})$, where $\sigma_{t-1}$ is a vector of standard deviations from {$\theta_{t-1}^{\left( i \right)}\}$ (a nine element vector with the standard deviation for each of the 9 parameters from $\theta$).
2. Simulate a candidate retrospective cohort ($x^{*}$) using our simulation model, $f$: $x^{*}\sim f(x|\theta^{**}$).
3. Include $\theta^{**}$ in {$\theta_{t}^{\left( i \right)}\}$ if $\pi(\theta^{**}$) and summary statistics of $x^{*}$ fulfill a set of criteria $C_{t}$ (S2 Table). Define the number of particles included in {$\theta_{t}^{\left( i \right)}\}$ as $n_{t}$.
4. Calculate the weight for all particles $\theta_{t}^{\left( i \right)}$in {$\theta_{t}^{\left( i \right)}\}$ as

$$w_{t}^{(i)}=\left\{ \begin{matrix} 1,\text{ if }\text{t}\text{ = 0} \\ \frac{\pi(\theta_{t}^{\left( i \right)})}{\sum_{j=1}^{n_{t-1}} w_{t-1}^{\left( j \right)}K_{t}(\theta_{t-1}^{\left( j \right)},\theta_{t}^{\left( i \right)})}\text{, if t>0} \end{matrix} \right.$$

5. Normalize the weights $w_{t}^{(i)}$ so that they sum to 1.
6. While $t<T$, set $t=t+1$ and go back to step (2).
7. Take {$\theta_{T}^{\left( i \right)}\}$ as the posterior distribution of $\theta$.

Along with a description of our model, $f(x|\theta^{**}$), and our threshold criteria $C_{t}$, this is a full description of our ABC-SMC algorithm. Our model generates 4,875 couple relationship histories (the size of the entire Rakai couple cohort [2]) from our copula model (Section IV) and then runs our couple transmission model with $\theta^{**}$ as input we to produce 4,875 couple serostate time series. We then generated an “observed couple cohort” from these time series by “observing” couples every 10 months from 1994-1999 with observations right-censored based on loss to follow-up as explained above (Methods in main text). We then selected a retrospective cohort from this cohort and grouped couples into incident, prevalent and late couples following the methods in Wawer et al. (Methods in main text), yielding the data set $x^{*}\sim f(x|\theta^{**}$).

We now describe the threshold criteria $C_{t}$, which are also given in S2 Table. The number of couples in each of the three couple groupings ($N_{\mathrm{incident}}^{*},N_{\mathrm{prevalent}}^{*},N_{\mathrm{late}}^{*})$ in $x^{*}$ is not constrained by the model to match those in the Rakai retrospective cohort ($N_{\mathrm{incident}}=23,$ $N_{\mathrm{prevalent}}=161,$ $N_{\mathrm{late}}=51)$. Our first threshold criteria are to loosely restrict ($N_{\mathrm{incident}}^{*},N_{\mathrm{prevalent}}^{*})$ so that they are similar to ($N_{\mathrm{incident}},$ $N_{\mathrm{prevalent}})$, with increasing similarity required as $t$ increases. We do not restrict $N_{\mathrm{late}}^{*}$ because all late phase parameters are fixed in our fitting procedure, and the focus of this analysis is the elevated infectivity of the acute phase relative to the chronic phase. Restrictions on ($N_{\mathrm{incident}}^{*},N_{\mathrm{prevalent}}^{*})$ ensured that the pre-couple and extra-couple transmission parameters chosen were approximately of the right magnitude to generate that many couples in each of these two groupings.

Because the small sample sizes in each couple grouping must be accounted for when fitting our simulations to the data, we sampled exactly 23 incident couples and 161 prevalent couples from $x^{*}$, and only considered these couples in all subsequent threshold criteria. For these couples, we constructed a table of the same format as given in Wawer et al. Table 1 (S4 Table), where the number of secondary partners that seroconverted in each observation interval is recorded:

| Incident Couples | | | Prevalent Couples | | |
| --- | --- | --- | --- | --- | --- |
| Interval | Infected | Not Infected | Interval | Infected | Not Infected |
| 1 | I_I1_ | S_I1_ | 1 | I_P1_ | S_P1_ |
| 2 | I_I2_ | S_I2_ | 2 | I_P2_ | S_P2_ |
| 3 | I_I3_ | S_I3_ | 3 | I_P3_ | S_P3_ |
| 4 | I_I4_ | S_I4_ | 4 | I_P4_ | S_P4_ |

Because of the subsampling described above, we note that I_I1_ + S_I1_ = 23 and I_P1_ + S_P1_ = 161, though subsequent row sums were not restricted and varied based on random variation in loss to follow-up, with loss to follow-up rates parameterized by empirical data as descried in Section I. For each observation interval, we calculated a G test statistic, comparing the simulated data with the real Rakai retrospective cohort. For instance, the G statistic for the first observation interval 1 from incident couples was calculated from the table:

|  | Infected | Not Infected |
| --- | --- | --- |
| Simulation | I_I1_ | S_I1_ |
| Data | 10 | 13 |

G statistics for all eight observation intervals were then summed to yield $G^{*}$, which was used as a goodness of fit summary statistic. We only accepted $\theta^{**}$ if $G^{*}<G_{t}$, where $G_{t}$ were a set of decreasing threshold criteria chosen to increase model fit through subsequent ABC-SMC iterations.

While $G^{*}$ provides some indication of model fit, this statistic does not account for dependence between observation intervals. Thus, we also calculated $\hat{\lambda}_{\mathrm{prevalent}}^{*}=\frac{\sum_{k=1}^{4} I_{P,k}}{\sum_{k=1}^{4} I_{I,k}+S_{I,k}}$ as the mean hazard of the secondary partner seroconverting amongst all prevalent couples and only accepted $\theta^{**}$ if $\hat{\lambda}_{\mathrm{prevalent}}^{*}$ was near to $\hat{\lambda}_{\mathrm{prevalent}}=$ (i.e. the proportion of prevalent couple observation intervals in which the secondary partner seroconverted), as defined by $\hat{\lambda}_{\mathrm{prevalent}}^{*}\in(m_{t}^{-1}\hat{\lambda}_{\mathrm{prevalent}},{m_{t}\hat{\lambda}}_{\mathrm{prevalent}})$, where $m_{t}$ are a set of scalars between 0 and 1 that increase towards 1 through subsequent ABC-SMC iterations.

Unlike prevalent couples, which are all assumed to have an index partner in their chronic phase, the hazard experienced by secondary partners in incident couples varies between observation intervals and the mean hazard across incident couple intervals is therefore uninformative. We did, however, calculate RH_acute_ using the Poisson regression and person-time tabulating approach of Wawer et al. We first calculated the acute to chronic phase relative hazard in a univariate unadjusted Poisson regression where secondary partner seroconversion was regressed against couple group (RH^*^_acute,univariate_):

seroconversion ~ log(person-time at risk) + group

We only accepted $\theta^{**}$ if RH^*^_acute,univariate_ $\in(r_{t}^{-1}\mathrm{RH}_{acute, univariate},r_{t}\mathrm{RH}_{acute, univariate})$, where $r_{t}$ are a set of scalars between 0 and 1 that increase towards 1 through subsequent ABC-SMC iterations and $\mathrm{RH}_{acute, univariate}=11.0$ (Table 1 in the main text). This ensured that $\theta^{**}$ were accepted that ensured that the ratio between the hazard of first interval incident couples and prevalent couples matched that from the actual data.

Finally, we also calculated RH_acute_ in an “omniscient” Poisson regression where we assumed that the secondary partners’ risk covariates were assumed to have been perfectly observed (i.e. equivalent to measuring all sources of heterogeneity in risk perfectly), and were included in the regression on a log scale (RH_acute,omnitient_):

seroconversion ~ log(person-time at risk) + group + log(true risk deviate).

We then calculated $h^{*}=\frac{{\mathrm{RH}^{*}}_{acute,univariate}}{{\mathrm{RH}^{*}}_{acute,omnitient}}$, which indicates the ratio between the unadjusted and the fully adjusted hazard ratios. As shown in our simulation results, increasing $\sigma_{\mathrm{hazard}}$ increases $\mathrm{RH}_{acute,univariate}$ but by accounting for observed sources of heterogeneity, adjusted multivariate regression models can reduce the estimated relative hazard towards the true value (Fig. 7 in main text). From Wawer et al.’s analysis, the ratio between the univariate relative hazard and the partially adjusted relative hazard is $11.0/7.25$ (Table 1 in main text). This gives a lower bound for the ratio $h^{*}$ because we would expect the denominator to have been further decreased had Wawer et al. observed and adjusted for more sources of heterogeneity in risk. Thus, our final criteria for accepting $\theta^{**}$ was that $h^{*}>$ 11.0/7.25.

We ran this ABC-SMC with these summary statistic criteria for particle acceptance for 5 sequential Monte Carlo iterations. Across these five SMC batches, we simulated approximately 500,000 retrospective cohorts for a total of 390 node-days of computing time on the 12-core nodes of the Lonestar Linux Cluster of the Texas Advanced Computing Center. The approach of each intermediate distribution to the final posterior distribution is shown for acute phase and transmission parameters in S9 Fig.

**IX. Cited References**

1. Wawer MJ, Gray RH, Sewankambo NK, Serwadda D, Li XB, et al. (2005) Rates of HIV-1 transmission per coital act, by stage of HIV-1 infection, in Rakai, Uganda. J Infect Dis 191: 1403–1409. doi: 10.1086/429411.

2. Porter L, Hao L, Bishai D, Serwadda D, Wawer MJ, et al. (2004) HIV status and union dissolution in sub-Saharan Africa: the case of Rakai, Uganda. Demography 41: 465–482.

3. Grabowski MK, Lessler J, Nalugoda F, Reynolds SJ, Ssekubugu R, et al. (2014) Introduction of HIV into stable heterosexual couples in Rakai, Uganda before and after ART [abstract]. Conference on Retroviruses and Opportunistic Infections 2014; 3–6 Mar 2014; Boston, MA, US.

4. Bellan SE, Fiorella KJ, Melesse DY, Getz WM, Williams BG, et al. (2013) Extra-couple HIV transmission in sub-Saharan Africa: a mathematical modelling study of survey data. Lancet 6736: 1–9. doi: 10.1016/S0140-6736(12)61960-6.

5. UNAIDS (2013) Global Report: UNAIDS report on the global AIDS epidemic.

6. Time from HIV-1 seroconversion to AIDS and death before widespread use of highly-active antiretroviral therapy: a collaborative re-analysis. Collaborative Group on AIDS Incubation and HIV Survival including the CASCADE EU Concerted Action. Concerted Action on SeroConversion to AIDS and Death in Europe. Lancet 355: 1131-1137.

7. Hollingsworth TD, Anderson RM, Fraser C (2008) HIV-1 transmission, by stage of infection. J Infect Dis 198: 687–693. doi: 10.1086/590501.

8. Toni T, Welch D, Strelkowa N, Ipsen a., Stumpf MP. (2009) Approximate Bayesian computation scheme for parameter inference and model selection in dynamical systems. J R Soc Interface 6: 187–202. doi: 10.1098/rsif.2008.0172.
